# Supplementary material for: The Short Chain Fatty Acid Butyrate Imprints an Antimicrobial Program in Macrophages
Source: Immunity. 2019 Feb 19;50(2):432–445.e7. doi: 10.1016/j.immuni.2018.12.018 (PMC6382411; doi:10.1016/j.immuni.2018.12.018)
Supplement: Document S1. Figures S1–S6 [file mmc1.pdf]

Immunity, Volume *50*

Supplemental Information

The Short Chain Fatty Acid Butyrate Imprints an Antimicrobial Program in Macrophages

Julie Schulthess, Sumeet Pandey, Melania Capitani, Kevin C. Rue-Albrecht, Isabelle Arnold, Fanny Franchini, Agnieszka Chomka, Nicholas E. Ilott, Daniel G.W. Johnston, Elisabete Pires, James McCullagh, Stephen N. Sansom, Carolina V. Arancibia-Cárcamo, Holm H. Uhlig, and Fiona Powrie

Supplemental Figures

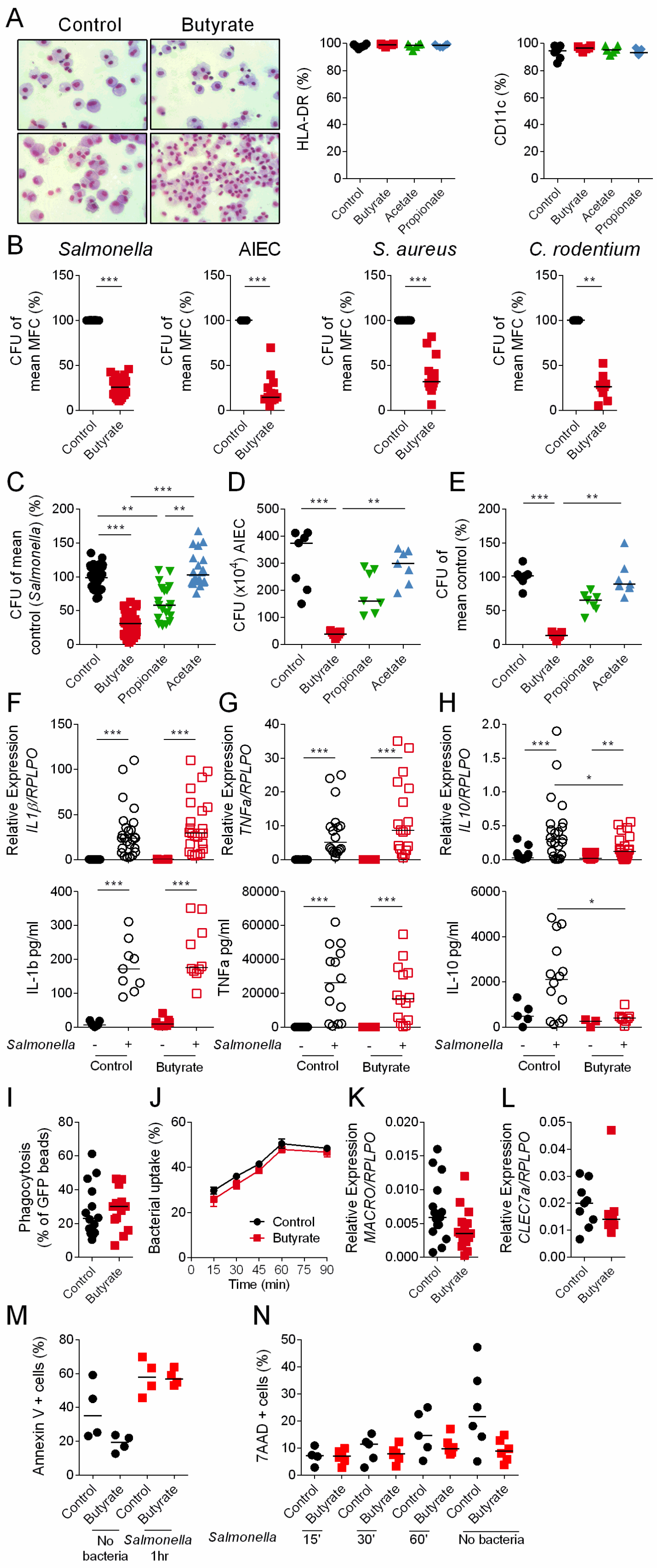

Figure S1

Figure S1: Increased antimicrobial activity by Mϕ differentiated in the presence of SCFA (Figure 1)

(A) Morphology and quantification of CD11c and HLA-DR expression on Control (left) and Butyrate (right) macrophages visualised by GIEMSA staining of cytospin preparations (X100) and FACS. Each dot represents one independent donor.

(B) Control and butyrate differentiated macrophages were infected for 1 hour with *Salmonella*, *AIEC*, *S. aureus* and *C. rodentium* followed by gentamicin treatment for 2 hours before cell lysis. Values are represented as % CFU of mean control macrophages.

(C-E) Gentamicin protection assay on Control, Butyrate, Acetate and Propionate differentiate macrophages infected with Salmonella (C) and (D) AIEC. Values are represented as % CFU of mean control macrophages or as absolute CFU count (E).

(F-H) Relative mRNA and protein expression of *IL1b* (F), *TNFa* (G) and *IL10* (H) by Control and Butyrate macrophages, at steady state and after 3 hours of *Salmonella* infection.

(I, J) Mϕ phagocytosis rates were assessed using FITC beads coated with human IgG on a flow cytometry-based assay and (J) bacterial uptake was assessed using non-opsonised GFP-*Salmonella* over 90 min.

(K-L) Relative mRNA expression of *MARCO* (K) and *CLEC7A* (L) in Control and Butyrate macrophages.

(M-N) Annexin V staining (n=4) (M) and 7AAD staining (N) in Control and Butyrate macrophages (n=4-6).

Each dot represents a healthy donor. Mann-Whitney U test was performed to investigate significant differences. Each dot represents one independent donor. Statistical significance was determined using Mann-Whitney U test *p<0.05, **p<0.01 and ***p<0.001.

Figure S2

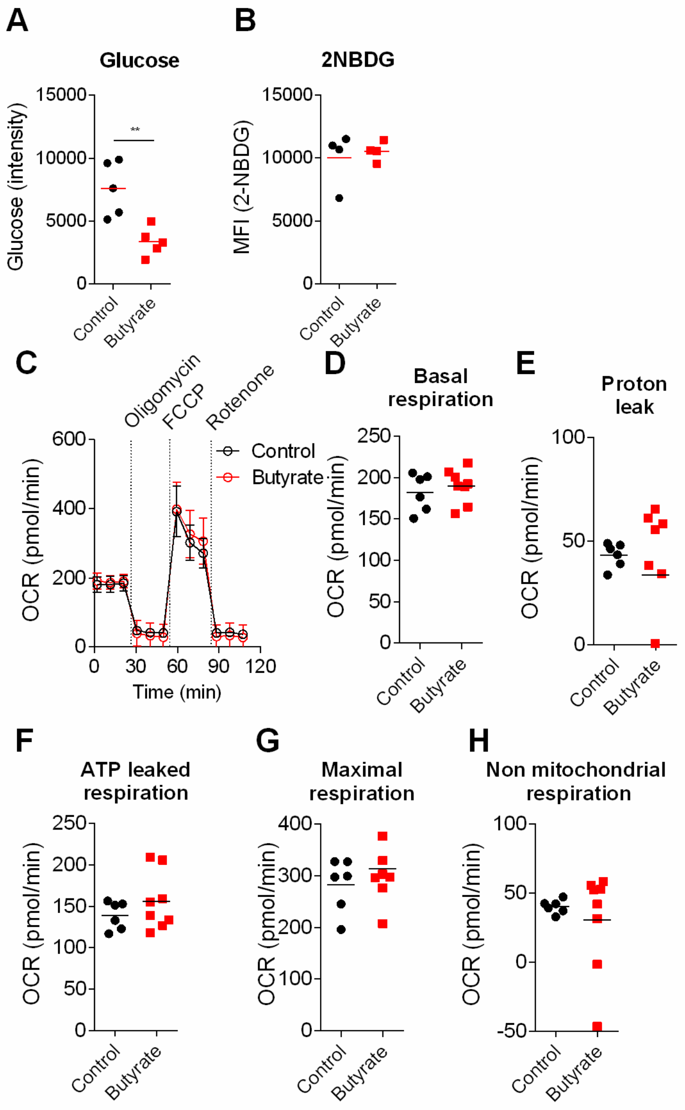

Figure S2: Oxygen consumption rate is similar in control and butyrate-treated macrophages (Figure 2)

(A) Intracellular glucose amounts were determined by Liquid chromatography /Mass Spectrometry (n=5).

(B) Uptake of 2-NBDG , a fluorescent glucose analog measured by FACS (n=4).

(C-H) Oxygen consumption rate of control and butyrate-treated macrophages after oligomycin, Carbonyl cyanide-*4*-(trifluoromethoxy) phenylhydrazone (FCCP) and rotenone treatment over time as determined by seahorse mito-stress test (C). Individual components of this assay including: Basal respiration (D), proton leak (E), adenosine tri-phosphate (ATP) leaked respiration (F), maximal respiration (G), Non mitochondrial respiration (H) is presented. Experiments were performed on each

donor with 8 technical replicates. Experiments with 3 different donors yielded similar results. Statistical significance was determined using **$p<0.01$; Mann-Whitney U test.

Figure S3

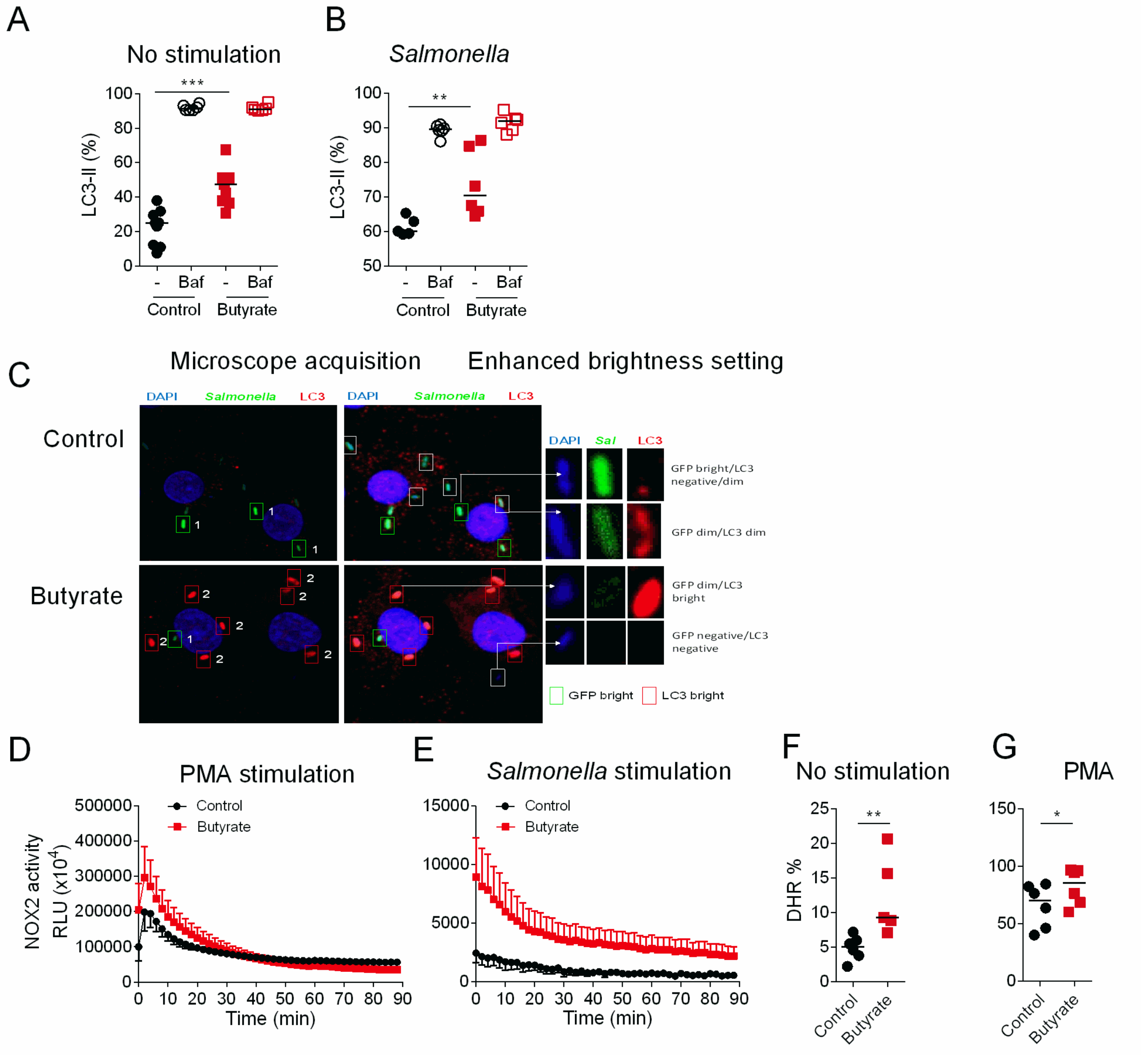

Figure S3 Increase of LC3 coated bacteria and ROS production in butyrate macrophages (Figure 2)

(A, B) Percentage LC3-II expression by flow cytometry at steady state (A) or after 1 hour infection with *Salmonella* (B) in Control and Butyrate macrophages. In the control conditions, cells are treated with 75nM of Bafilomycin A1 (baf) (positive control) for 2 hour before the LC3 staining.

(C) Representative confocal microscopy image of Control and Butyrate macrophages showing different stages of *Salmonella* degradation with increased LC3 coating in Butyrate macrophages (example quantification corresponding to the images provided in Fig. 2L). Control and Butyrate macrophages were infected with GFP-*Salmonella* and stained with LC3 and DAPI. For the microscopy quantification of *Salmonella* degradation, bacteria were classified into GFP^{bright} $LC3^{negative/dim}$ (i.e. early stage *Salmonella infection with intact gfp* signal) and $GFP^{dim}LC3^{bright}$ (LC3 coated *Salmonella* with quenched or degraded gfp signal). In addition there are intermediate stages of $GFP^{dim}LC3^{dim}$ (Intermediate phase)

and GFP^{negative}LC3 ^{negative} *Salmonella* (end stage degradation with bacterial DNA remnant). DAPI stain was used to identify intracellular bacteria. The microscopy acquisition setting was used to identify GFP^{bright} and LC3^{bright} bacteria whereas the enhanced brightness setting of each image was used to confirm the bacterial DNA content and to identify intermediate stages.

(D, E) ROS produced by NOX2 was quantified by a chemi-luminescence based L0-12 assay in Control and Butyrate macrophages stimulated with or without PMA (100ng/ml) (D) or infected by *Salmonella* at MOI 10 for 30 min (E) before evaluating NOX2 activity. Data represent the mean of 4 independent experiments from 3 donors.

(F, G) Intracellular reactive oxygen species evaluated with dihydrorhodamine (DHR) FACS in Control and Butyrate macrophages at steady state (F) or after PMA stimulation for 15 min (G). Each dot represents one independent donor.

Statistical significance was determined using Mann-Whitney U test *p<0.05, **p<0.01 and ***p<0.001.

Figure S4

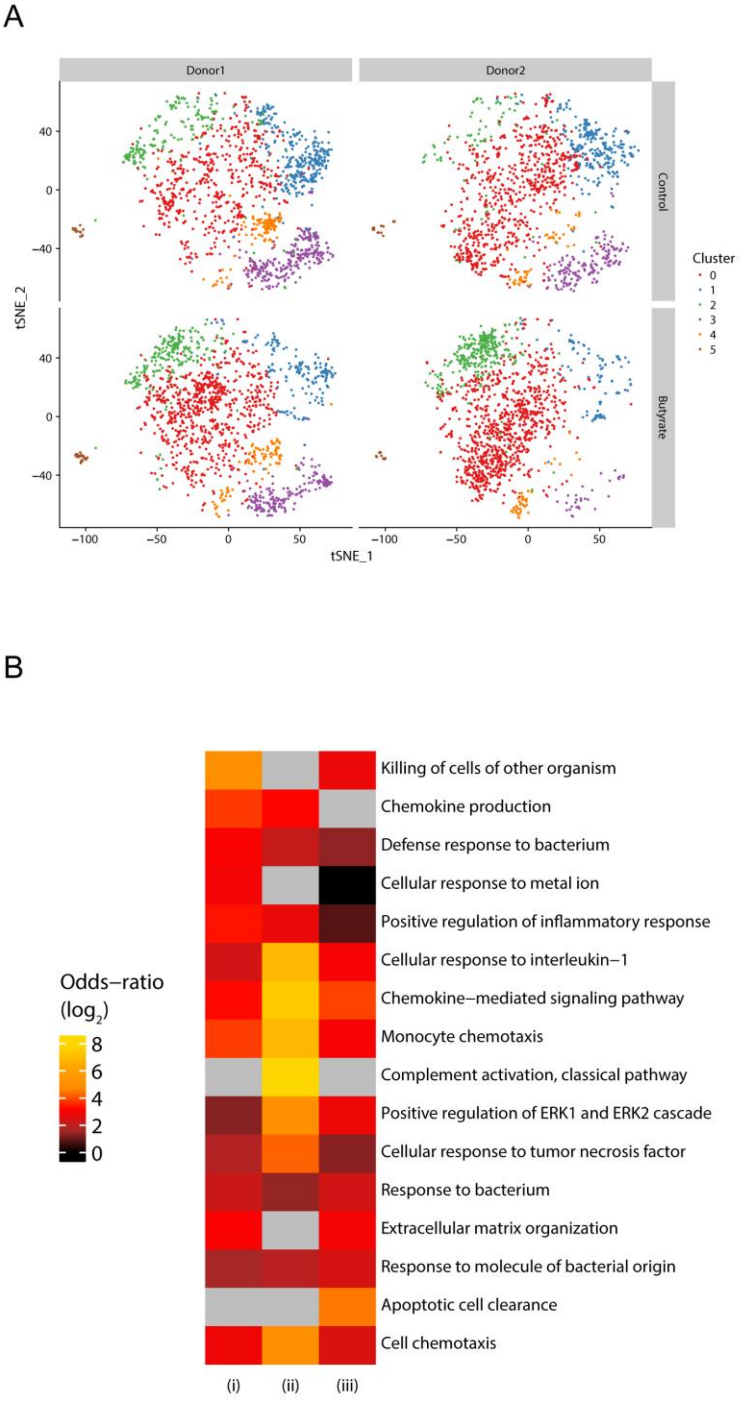

Figure S4: Impact of butyrate across subpopulations of differentiated macrophages (Figure 3)

(A) The t-SNE projection shown in Fig 4, deconvoluted to reveal the distribution of cells from the four

individual samples. A noticeable increase of cells in the lysosomal (cluster 0) and antimicrobial (cluster 2) compartments can be observed.

(B) Comparative heat map of $\log_2$ odds-ratio for over-representation of biological pathways (GO) in genes (i) commonly up-regulated by butyrate across the entire population of differentiated macrophages, (ii) specifically down-regulated in cluster 2 (antimicrobial Macrophages), and (iii) globally down-regulated by butyrate across the entire population of differentiated Macrophages.

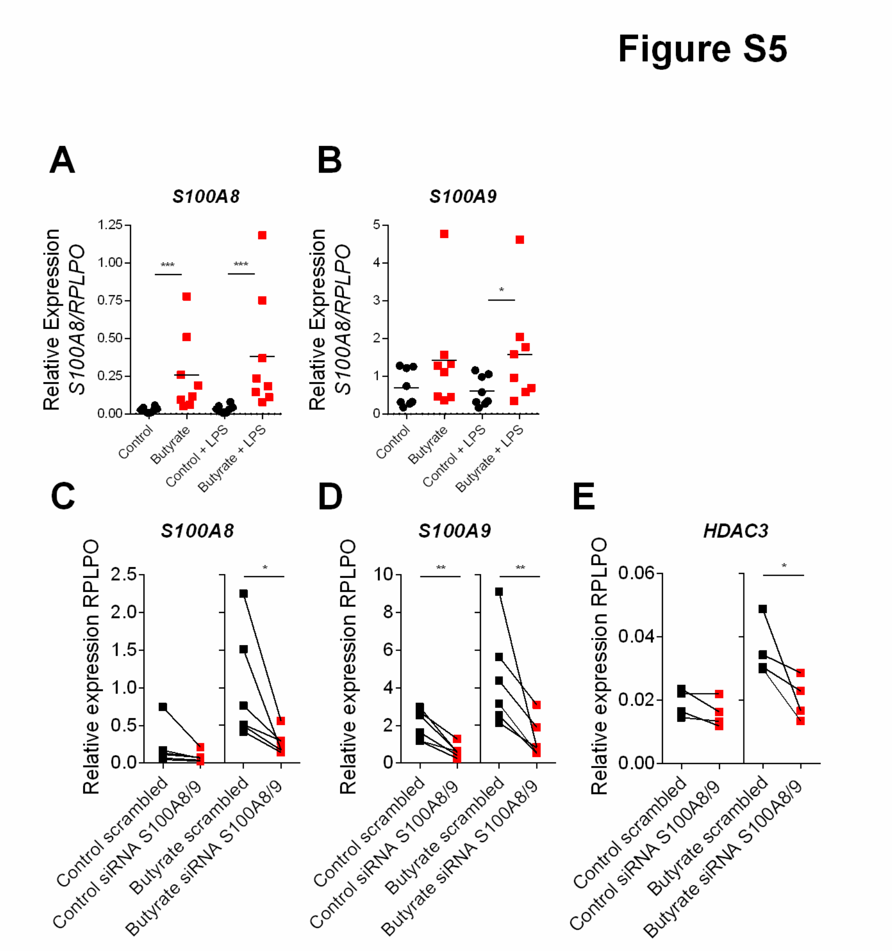

Figure S5: *S100A8*, *S100A9* and *HDAC3* mRNA expression in control and butyrate-treated macrophages. (Figure 4 and 5)

(A-B) *S100A8* (A) and *S100A9* (B) mRNA expression was measured by qPCR in control and butyrate-treated macrophages in the presence or absence of LPS. Each dot represents one donor (n=8).

(C-E) *S100A8* (C) *S100A9* (D) and HDAC3 (E) mRNA expression was measured by qPCR. Each dot represents one donors (n=6).

Statistical significance was determined using Mann-Whitney U test $*p<0.05$, $**p< 0.01$, $***p<0.001$.

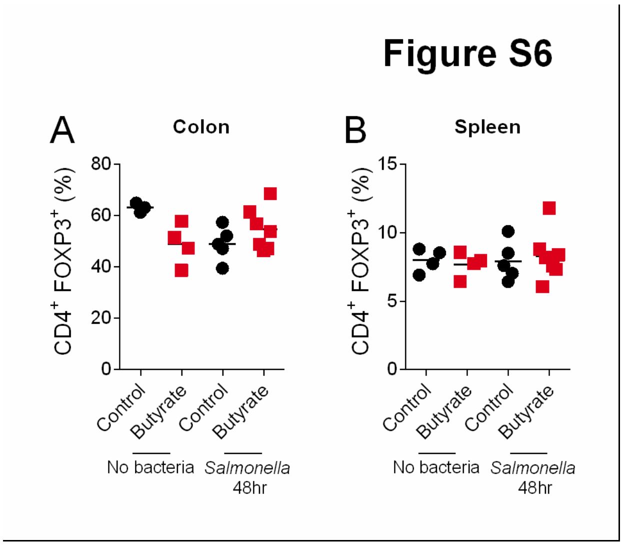

Figure S6: CD4$^+$FOXP3$^+$ cells do not show any change in Control and butyrate treated mice. (Figure 6)

(A-B) Percent of CD4$^+$FOXP3$^+$ cells in the colon (A) and (B) spleen. Colonic lamina propria and splenic CD45$^+$CD3$^+$CD4$^+$FOXP3$^+$ T cells analysed by flow cytometry in uninfected mice or 48hours after *Salmonella* infection. Butyrate-treated mice received butyrate 5 days before and during infection in the drinking water.

Supplementary Tables

Table S1: Cluster marker genes common to both control and butyrate macrophages

The table contains the positive and negative marker genes identified for each of the clusters of macrophages discovered by the single-cell RNA-sequencing analysis (Fig 3A). Only marker genes that achieved significance in all separate, per-sample tests (BH adjusted p-value < 0.05) are listed.

Table S2: Genes differentially expressed between between control and butyrate macrophages within each cluster of differentiated macrophages.

This table contains the lists of genes differentially expressed by between control and butyrate macrophages within each of the clusters of differentiated (non-cell cycle associated) macrophages (see Methods).

Table S3: Gene Ontology categories enriched in genes differentially expressed between control and butyrate macrophages

The table contains the details of the Gene Ontology (Biological Process) categories found to be signficantly enriched in each of the three groups of genes differentially expressed between control and butyrate macrophages (Fig 3E).
