# Supplementary material for: The Short Chain Fatty Acid Butyrate Imprints an Antimicrobial Program in Macrophages
Source: Immunity. 2019 Feb 19;50(2):432–445.e7. doi: 10.1016/j.immuni.2018.12.018 (PMC6382411; doi:10.1016/j.immuni.2018.12.018)
Supplement: Document S2. Article plus Supplemental Information [file mmc5.pdf]

Immunity

Article

The Short Chain Fatty Acid Butyrate Imprints an Antimicrobial Program in Macrophages

Graphical Abstract

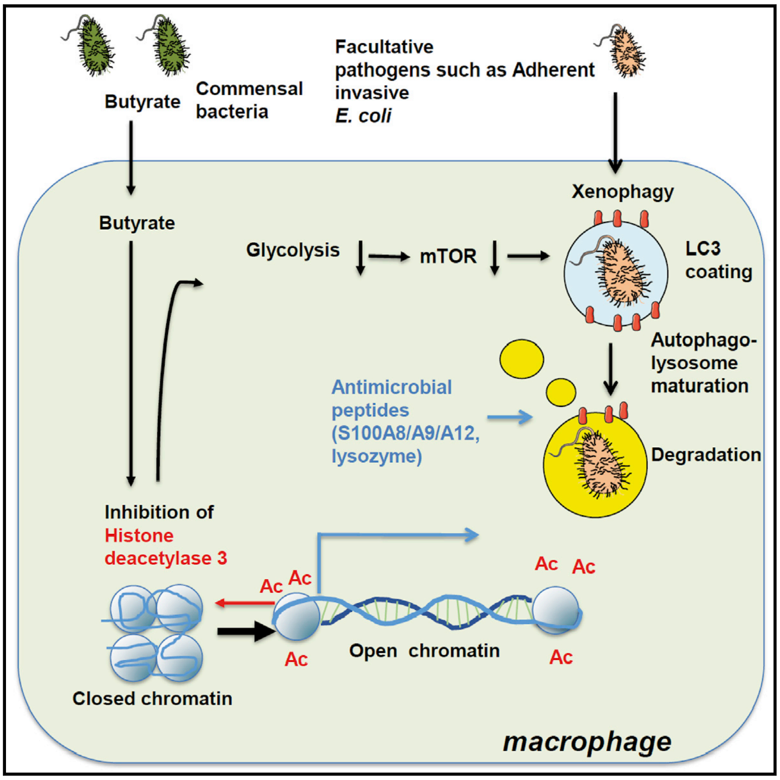

Highlights

- Butyrate induces differentiation of macrophages with potent antimicrobial function
- Enhanced antimicrobial function is a consequence of glycolysis and mTOR inhibition
- Single-cell RNA-sequencing identifies butyrate-induced antimicrobial peptides
- Butyrate inhibits HDAC3 to drive metabolic changes and microbicidal function

Authors

Julie Schulthess, Sumeet Pandey, Melania Capitani, ..., Carolina V. Arancibia-Cárcamo, Holm H. Uhlig, Fiona Powrie

Correspondence

fiona.powrie@kennedy.ox.ac.uk

In Brief

Macrophages maintain gut homeostasis by eliminating invasive pathogens and regulating inflammatory responses. Schulthess et al. demonstrate that butyrate, a bacterial fermentation product, imprints potent antimicrobial activity during macrophage differentiation through HDAC3i function.

Schulthess et al., 2019, Immunity *50*, 432–445
February 19, 2019 © 2018 The Authors. Published by Elsevier Inc.
https://doi.org/10.1016/j.immuni.2018.12.018

CellPress

Immunity
Article

The Short Chain Fatty Acid Butyrate Imprints an Antimicrobial Program in Macrophages

Julie Schulthess,[1,2,5] Sumeet Pandey,[2,5] Melania Capitani,[2] Kevin C. Rue-Albrecht,[1] Isabelle Arnold,[2] Fanny Franchini,[1] Agnieszka Chomka,[1] Nicholas E. Ilott,[1] Daniel G.W. Johnston,[1] Elisabete Pires,[3] James McCullagh,[3] Stephen N. Sansom,[1,6] Carolina V. Arancibia-Cárcamo,[2,6] Holm H. Uhlig,[2,4,7] and Fiona Powrie[1,2,7,8,*]

[1]Kennedy Institute of Rheumatology, Nuffield Department of Orthopaedics, Rheumatology and Musculoskeletal Sciences, University of Oxford, Roosevelt Drive, OX3 7FY, UK

[2]Translational Gastroenterology Unit, Experimental Medicine Division, Nuffield Department of Medicine, University of Oxford, John Radcliffe Hospital, Oxford OX3 9DU, UK

[3]Chemistry Research Laboratory, Department of Chemistry, University of Oxford, Mansfield Road, Oxford OX1 3TA, UK

[4]Department of Pediatrics, University of Oxford, John Radcliffe Hospital, Oxford OX3 9DU, UK

[5]These authors contributed equally

[6]These authors contributed equally

[7]These authors contributed equally

[8]Lead contact

*Correspondence: fiona.powrie@kennedy.ox.ac.uk

https://doi.org/10.1016/j.immuni.2018.12.018

SUMMARY

Host microbial cross-talk is essential to maintain intestinal homeostasis. However, maladaptation of this response through microbial dysbiosis or defective host defense toward invasive intestinal bacteria can result in chronic inflammation. We have shown that macrophages differentiated in the presence of the bacterial metabolite butyrate display enhanced antimicrobial activity. Butyrate-induced antimicrobial activity was associated with a shift in macrophage metabolism, a reduction in mTOR kinase activity, increased LC3-associated host defense and antimicrobial peptide production in the absence of an increased inflammatory cytokine response. Butyrate drove this monocyte to macrophage differentiation program through histone deacetylase 3 (HDAC3) inhibition. Administration of butyrate induced antimicrobial activity in intestinal macrophages *in vivo* and increased resistance to enteropathogens. Our data suggest that (1) increased intestinal butyrate might represent a strategy to bolster host defense without tissue damaging inflammation and (2) that pharmacological HDAC3 inhibition might drive selective macrophage functions toward antimicrobial host defense.

INTRODUCTION

The gastrointestinal tract is colonized by a high density of commensal bacteria and is a major site of pathogen entry (Rooks and Garrett, 2016) requiring robust barrier function. Short chain fatty acids (SCFAs) are derived from bacterial fermentation of dietary fibers in the colonic lumen. The SCFAs butyrate, propionate, and acetate promote intestinal epithelial barrier function and regulate the host mucosal immune system (Vinolo et al., 2011b). For example, butyrate serves as a primary energy source for intestinal epithelial cells, the first line of cellular defense against invading pathogens. Butyrate also regulates stem cell turnover in intestinal epithelial crypts (Kaiko et al., 2016). SCFAs, and in particular butyrate also promote regulatory T cells (Treg) in the colon by inhibiting histone deacetylase (HDAC) activity at the *Foxp3* locus (Arpaia et al., 2013; Furusawa et al., 2013; Smith et al., 2013). Furthermore, exposure of peripheral blood mononuclear cells such as neutrophils, macrophages, and dendritic cells to SCFAs or other HDAC inhibitors, such as trichostatin (TSA), inhibits inflammatory cytokine production (Chang et al., 2014; Usami et al., 2008; Vinolo et al., 2011a).

Mouse models of intestinal inflammation suggest that butyrate plays an immune regulatory role *in vivo* (Furusawa et al., 2013). This is potentially relevant for human immunopathology since reduced numbers of butyrate-producing bacteria were found in the gut mucosa and in fecal samples from patients with inflammatory bowel disease (IBD) or colon cancer (Frank et al., 2007; Wang et al., 2012).

Intestinal phagocytes, and tissue-resident macrophages in particular, act as an innate barrier in the intestine by clearing invading bacteria. Malfunctioning of this pathway is involved in the pathogenesis of IBD since defective microbicidal responses were identified in polygenic and monogenic forms of IBD (Peloquin et al., 2016; Uhlig and Powrie, 2018). In contrast to macrophages found in other organs, intestinal macrophages are largely replenished from blood monocytes (Bain et al., 2014). Thus, circulating monocytes enter the gut and undergo final differentiation in the lamina propria to become mature, highly phagocytic macrophages capable of bactericidal activity via mechanisms such as NADPH-oxidase-derived reactive oxygen species (ROS) and antimicrobial peptides and proteins (Bain et al., 2014; Smythies et al., 2005; Varol et al., 2009). The bacterial pathways that shape macrophage host defense in the intestine are poorly understood. Here we have investigated the ability of SCFAs to influence macrophage function. We show that SCFAs induce metabolic and transcriptional changes in macrophages, which enhances their bactericidal functions.

 © 2018 The Authors. Published by Elsevier Inc.
This is an open access article under the CC BY license (http://creativecommons.org/licenses/by/4.0/).

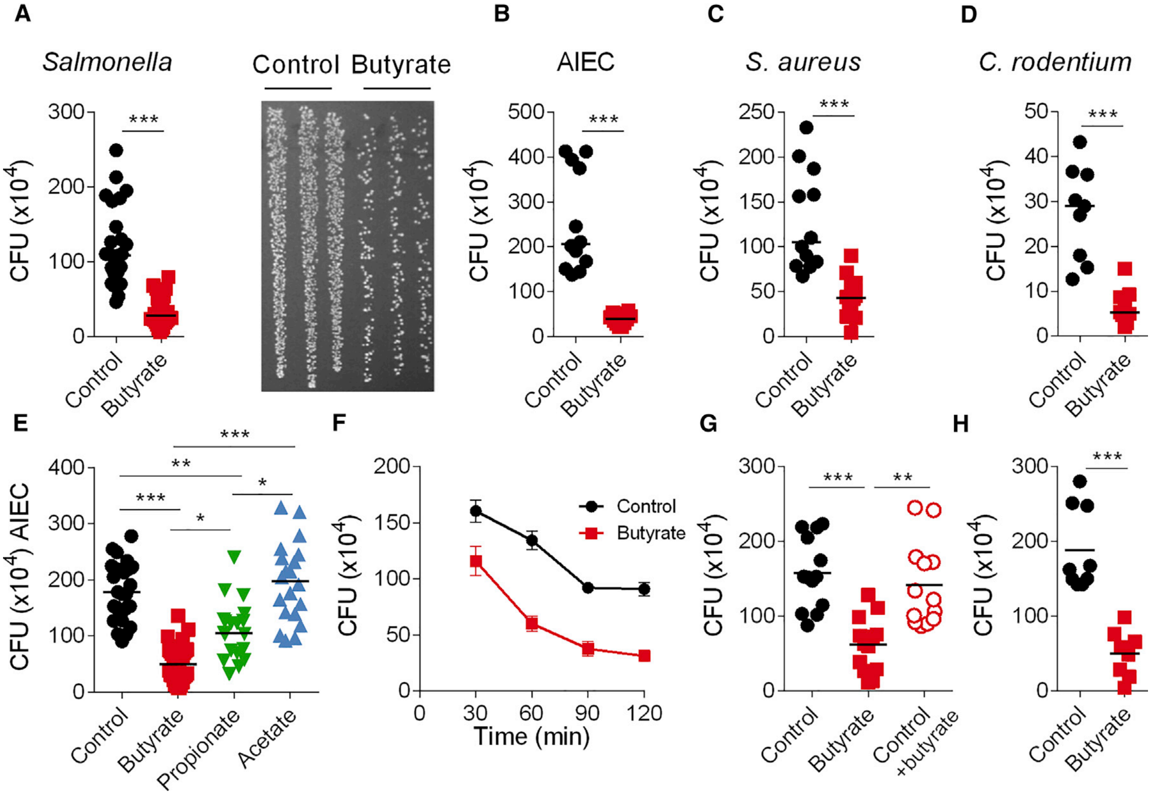

Figure 1. Increased Antimicrobial Activity by Macrophages Differentiated in Presence of Butyrate

(A–H) Gentamicin protection assay on control macrophages and butyrate macrophages with a range of different bacteria. Macrophages were infected for 1 h with *Salmonella enterica* serovar Typhimurium (*Salmonella*) (A, E–H), *adherent-invasive Escherichia coli* (AIEC) (B), *Staphylococcus aureus* (*S. aureus*) (C) or *Citrobacter rodentium* (*C. rodentium*; D) followed by gentamicin treatment for 2 h before cell lysis. Values represent absolute CFU counts. Each dot is representative of one donor. Representative image of agar plate showing *Salmonella* CFU (A, right).

(E) Gentamicin protection assay on macrophages treated with different SCFAs.

(F) Kinetics of elimination of *Salmonella* by control macrophages and butyrate macrophages.

(G) Short-term butyrate treatment: macrophages were treated for 3 h with butyrate prior to the gentamicin protection assay.

(H) Butyrate macrophages were cultured in the absence of butyrate for 24 h prior to the gentamicin protection assay.

Each dot represents one independent donor, experiments were repeated 3–8 times. Statistical significance was determined using Mann-Whitney U test $^{*}p < 0.05$, $^{**}p < 0.01$, and $^{***}p < 0.001$.

Please also see Figure S1.

RESULTS

Butyrate Exposure during Macrophage Differentiation Enhances Antimicrobial Activity

To assess the impact of SCFAs on human macrophages, we differentiated peripheral blood-derived $CD14^{+}$ monocytes with macrophage colony-stimulating factor (M-CSF) in the absence (control macrophages) or presence of butyrate (butyrate macrophages), propionate (propionate macrophages), or acetate (acetate macrophages). The presence of SCFAs during macrophage differentiation did not affect key macrophage characteristics such as morphology and surface expression of CD11c and HLA-DR (Figures S1A and S1B). However, SCFAs did affect the antimicrobial function of macrophages assessed in a gentamicin protection assay using a range of bacteria including gram negative (*Salmonella enterica* serovar Typhimurium, later on referred to as *Salmonella*); Crohn's disease-associated adherent-invasive *Eschericha coli* (*AIEC*), and *Citrobacter rodentium* (*C. rodentium*) and gram-positive *Staphylococcus aureus* (*S. aureus*) microbes (Figures 1A–1D and Figure S1B). As the anti-microbial effect was strongest with butyrate as opposed to proprionate and not observed with acetate, butyrate was studied further (Figure 1E and Figures S1C–S1E).

We next investigated the kinetics of antibacterial activity, cytokine secretion, phagocytic capacity, and apoptosis in control macrophages and butyrate macrophages. Butyrate macrophages displayed significantly increased elimination of intracellular *Salmonella* as early as 30 min after infection, and this was maintained over the 3 h duration of the gentamicin protection assay (Figure 1F). Despite altered bacterial clearance, control macrophages and butyrate macrophages displayed similar expression of *IL1B* and *TNF* mRNA and protein following 3 h of *Salmonella* infection (Figures S1F and S1G), whereas *IL10* expression was reduced in butyrate macrophages (Figure S1H). In contrast to the long-term exposure of butyrate during the differentiation process, incubation of control macrophages with butyrate for 3 h prior to the gentamicin protection assay did not induce a significant reduction in CFU compared to untreated

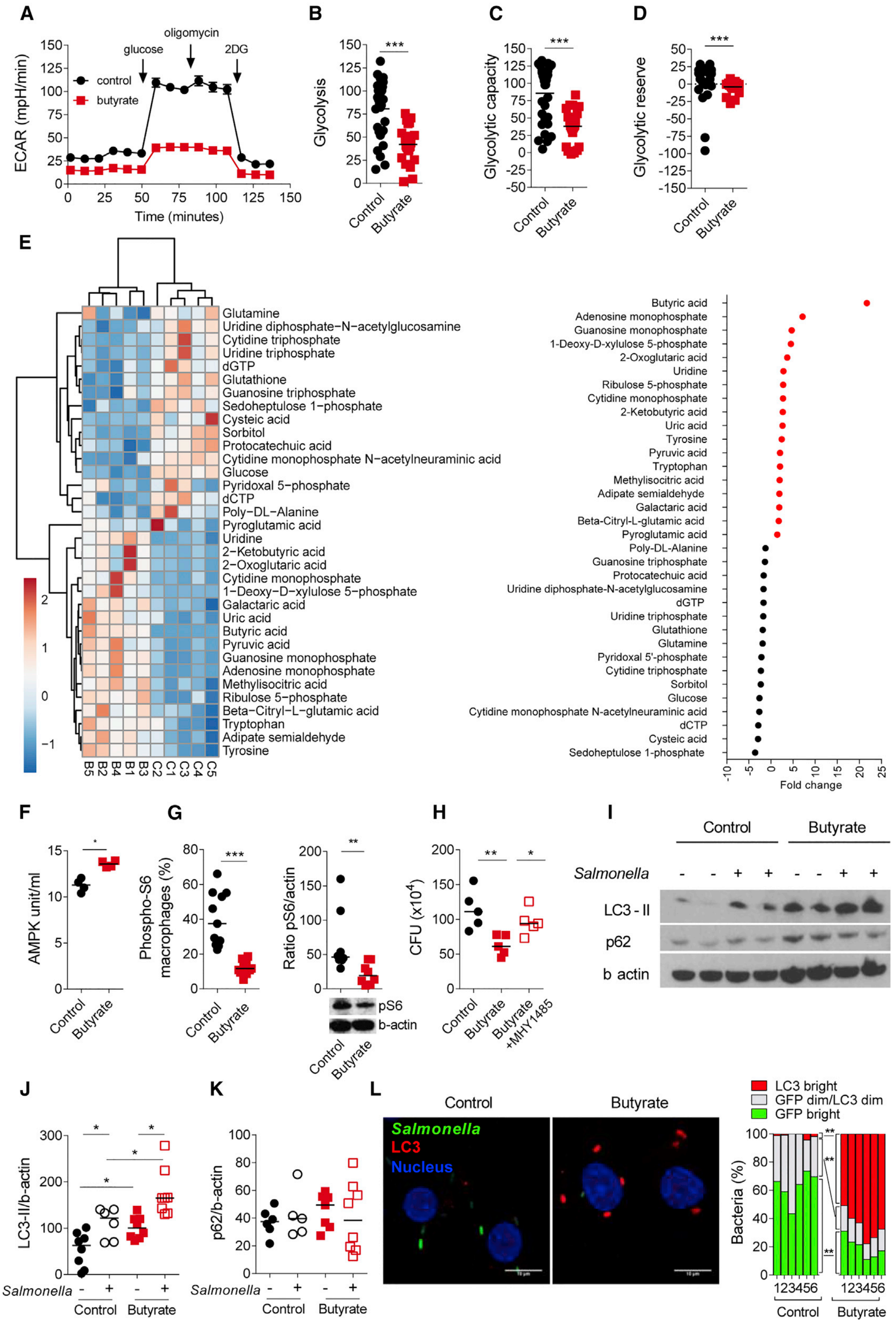

Figure 2. Increase of Antimicrobial LC3-Associated Immune Defense in Butyrate Macrophages

(A) Extracellular acidification rate (ECAR) measured at steady state in control and butyrate macrophages. Data represent the mean of nine biological replicates from three independent experiments.

(legend continued on next page)

control macrophages (Figure 1G). After 24 h incubation in butyrate-free media, butyrate macrophages retained enhanced antimicrobial activity, indicating that this response does not require butyrate at the time of infection (Figure 1H). The reduced bacterial load was not due to reduced phagocytosis as butyrate macrophages showed normal ingestion of FITC beads coated with human IgG and uptake of non-opsonised GFP-*Salmonella* over 90 min. In addition, they showed similar mRNA expression of the phagocytic receptors *MARCO* and *CLEC7A* compared to control (Figures S1I–S1L). Reduced numbers of bacteria in butyrate macrophages was not a consequence of increased cell death as the percentage of apoptotic cells was also similar between butyrate macrophages and control macrophages (Figures S1M and S1N). Taken together, these results show that the presence of butyrate during the differentiation of macrophages induces long-lasting antimicrobial activity without affecting phagocytosis, inflammatory cytokine production, or apoptosis.

Butyrate Alters Metabolism and Induces mTOR Dependent LC3-Associated Antimicrobial Clearance in Macrophages

As butyrate is a key energy source for epithelial cells (Vinolo et al., 2011b) and relevant amounts of butyrate are likely present in the lamina propria as suggested by portal vein concentrations (van der Beek et al., 2015), we tested whether butyrate exposure alters macrophage metabolism. Butyrate macrophages showed a decreased extracellular acidification rate (ECAR) in comparison to control macrophages (Figure 2A). Glycolysis, glycolytic capacity and glycolytic reserve were also significantly reduced in butyrate macrophages (Figures 2B–2D). To explore the metabolic pathways modulated by butyrate, we performed a metabolomic analysis by liquid chromatography–mass spectrometry. This confirmed substantial changes in the glycolysis pathway including a reduced glucose concentration (Figure 2E and Figure S2A). This is unlikely due to reduced glucose uptake as 2NBDG uptake in control and butyrate macrophages was similar (Figure S2B). Since we found similar mitochondrial oxidative phosphorylation (respiration, proton leak, oxygen consumption rate) between control and butyrate macrophages, the switch in glucose metabolism was not compensated for by increased mitochondrial energy metabolism (Figures S2C–S2H).

We found 34 significantly differentially expressed metabolites between control and butyrate macrophages (Figure 2E). Butyrate was the most differentially expressed metabolite in butyrate macrophages confirming the validity of this approach. Butyrate macrophages also contained higher amounts of adenosine monophosphate (AMP) a known inducer of AMP kinase (AMPK) that inhibits mTOR (Inoki et al., 2003) the master regulator of autophagy (Kim and Guan, 2015). In agreement with this hypothesis, AMPK Thr172 phosphorylation was significantly increased in butyrate macrophages compared to controls (Figure 2F). To test whether butyrate treatment blocked mTOR activity, we analyzed ribosomal protein S6 kinase phosphorylation by flow cytometry and immunoblot as a surrogate marker of mTOR activation. Indeed, butyrate macrophages showed a marked reduction of pS6 phosphorylation compared to control macrophages (Figure 2G). In support of the hypothesis that butyrate-induced antibacterial clearance depends on mTOR inhibition, we found that treatment with MHY1485, an mTOR activator (Figure 2H) blunted butyrate macrophage antibacterial activity (Choi et al., 2012).

Since mTOR is a key regulator of autophagy and autophagy-related processes, we tested whether butyrate-induced antimicrobial activity was associated with bacterial-associated autophagy protein microtubule-associated protein 1 light chain 3 alpha (LC3). We tested the LC3-I to LC3-II conversion by immunoblot, LC3 flow cytometry and confocal microscopy. Immunoblot indicated that the basal and *Salmonella* infection-induced LC3-II turnover was significantly increased in butyrate macrophages (Figure 2I and 2J). In contrast to the LC3-II increase, P62 protein (SQSTM1) expression was unchanged between control and butyrate macrophages (Figure 2I and 2K), indicating an overall functional degradation of the autophagosome and normal autophagic flux. We also confirmed the increase in LC3-II turnover quantitatively by flow cytometry (Figures S3A and S3B).

Next, we investigated whether the increase in LC3-II turnover induced in butyrate macrophages is localized to intracellular *Salmonella*. To test this hypothesis, we performed confocal imaging by staining LC3 in control and butyrate macrophages infected with GFP-*Salmonella* (Figure 2L). Viable intracellular bacteria expressed a strong GFP signal. The degradation process was characterized by subsequent LC3 coating and loss of the GFP signal. Whereas in control macrophages a large proportion of *Salmonella* expressed a bright GFP signal, we found that in butyrate macrophages bacteria expressed a significantly lower GFP bright signal and a higher percentage of *Salmonella* are associated with strong LC3 coating (Figure 2L, details of the microscopy approach are described in detail in Figure S3C).

(B–D) Quantification of glycolysis (B), glycolytic capacity (C) and glycolytic reserve (D).

(E) Heatmap of metabolites that were significantly higher or lower in control and butyrate macrophages as detected by mass-spectrometry (left panel). Results are from five healthy donors. Right panel: Fold change of all significantly higher or lower metabolites in control macrophages (black closed circles) and butyrate macrophages (red closed circles).

(F) AMPK phosphorylation (Thr172) measured by ELISA (n = 4 individual donors).

(G) Percentage of pS6 (left) and representative blot of phosphorylation and quantification (right) of the ribosomal protein S6 and β-actin in control and butyrate macrophages at steady state.

(H) Gentamicin protection assay performed on control macrophages, butyrate macrophages, and butyrate macrophages treated for 2 h with the mTOR activator MHY1485 (20 mM).

(I) Representative immunoblot of the expression of LC3-II, P62, and β-actin at steady state or after 2 h infection with *Salmonella*.

(J and K) Protein quantification performed by ImageJ of LC3-II (I) and P62 (J) compared to β-actin.

(L) Degradation of GFP-*Salmonella* and LC3 induction was assessed by confocal microscopy. Representative images and quantification of GFP fluorescence and LC3 accumulation as outlined in Figure S7C. Data from six independent donors in two independent experiments. Scale bar 5 μm. Each dot represents one donor. Statistical significance was determined using Mann-Whitney U test *p < 0.05, **p < 0.01, and ***p < 0.001.

Please also see Figures S2 and S3.

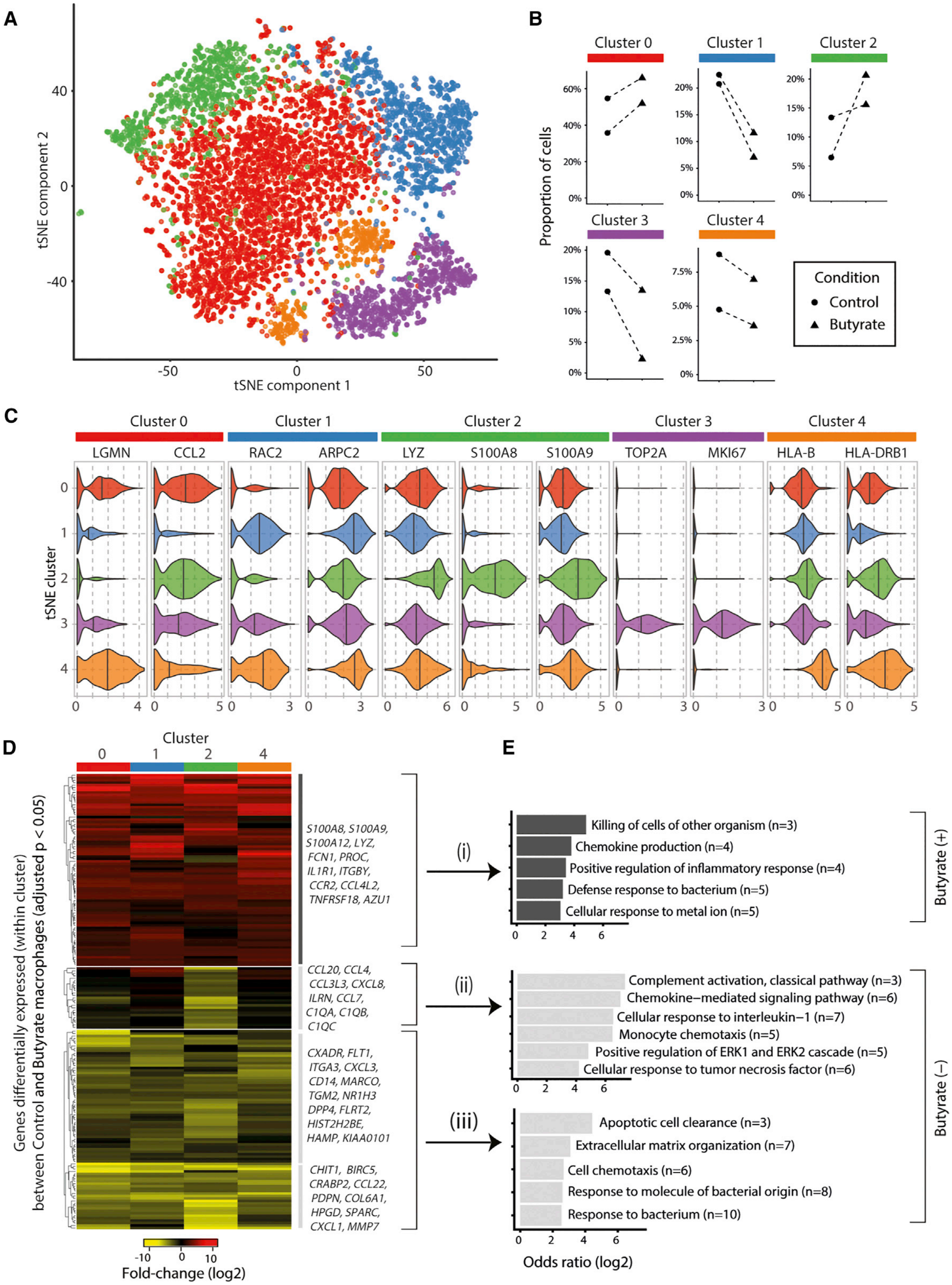

Figure 3. Butyrate Exposure Promotes an Antimicrobial Macrophage Phenotype

(A) Single-cell RNA-seq analysis of control and butyrate treated macrophages (n = 5,836 cells) from two healthy donors. The t-SNE projection shows the five subpopulations of macrophages that were identified with a graph-based clustering algorithm (see Methods).

(B) Quantitation of the proportions of control and butyrate macrophages ascribed to each of the sub-populations. Dashed lines indicate the sample pairs (shared donor identity).

(legend continued on next page)

Moreover, we investigated the role of ROS generated by NADPH oxidase activity, an anti-microbial effector mechanism that typifies LC3-associated phagocytosis. In macrophages, the activity of the NADPH oxidase 2 (NOX2) enzyme complex is required for efficient recruitment of LC3 to phagosomes restricting bacterial colonization, growth in the cytosol, and elimination of invading bacteria (Huang et al., 2009). We measured NADPH oxidase activity by luminol-chemiluminescence on macrophages stimulated with phorbol 12-myristate 13-acetate (PMA) (Figure S3D) or after *Salmonella* infection (Figure S3E). In both conditions, butyrate macrophages produced significantly more reactive oxygen species (ROS) as measured by a dihydrorhodamine (DHR) assay after PMA stimulation or *Salmonella* infection compared to controls (Figures S3F and S3G).

Calprotectin Mediates the Enhanced Antimicrobial Function of Butyrate Macrophages

The antimicrobial effects of butyrate depended on sustained exposure during macrophage differentiation. Since butyrate has a well-known role as an HDACi, we reasoned that its effect on macrophage differentiation may be a consequence of epigenetic changes and gene expression affecting the heterogeneity of the resulting macrophage population. We therefore employed single-cell RNA-sequencing, a method that facilitates the identification and transcriptomic characterization of previously unknown cell subtypes and states (Villani et al., 2017). We performed droplet-based single-cell RNA-seq analysis of control and butyrate macrophages from two healthy donors, retaining 5,981 cells for detailed analysis. We first performed an alignment between control and butyrate macrophages using an approach based on canonical correlation analysis (CCA) (Butler and Satija, 2018). We were able to align 98.7% of the cells, confirming the existence of broadly similar cell-states in both the control and butyrate macrophage populations. We identified five major clusters of macrophages (Figure 3A) and characterized these subpopulations by identifying marker genes for each cluster that were conserved between control and butyrate macrophages (selected examples shown in Figure 3C, and Table S1). The different clusters of macrophages were associated with distinct gene-expression profiles (cluster 0 lysosomal function; cluster 1 regulation of actin cytoskeleton and phagocytosis; cluster 4 phagosomal activity and antigen presentation; cluster 3 cell-cycle-associated genes). Cells in cluster 2 were characterized by genes associated with antimicrobial responses (*e.g., S100A8, S100A9)* and lysosomal functions (*LYZ*). Butyrate consistently increased the numbers of macrophages associated with the lysosomal (cluster 0) and antimicrobial phenotype (cluster 2) (Figure 3B).

To examine the influence of butyrate, we identified genes that were differentially expressed between control and butyrate macrophages within each of the clusters of differentiated (i.e., non-cell-cycle-associated) macrophages (see Methods). We found that butyrate predominantly induced changes in gene expression across the entire population of differentiated macrophages (Figure 3D, Table S2). Genes upregulated by butyrate were enriched for gene-ontology (GO) categories including "killing of cells of other organism" and "defense response to bacterium" (Figure 3E, Figure S4, Table S3). Butyrate downregulated genes associated with "cellular response to interleukin-1" and the classic complement (i.e., *C1QA*, *C1QB*, *C1QC*) activation genes in cells of cluster 2.

Overall, the single-cell data revealed a butyrate-induced antimicrobial signature characterized by the expression of *S100A8*, *S100A9*, *S10012*, *LYZ*, and *FCN1* particularly in the cluster 2 subset of differentiated macrophages (Figure 4A and S8). Because these data are based on only two individuals, we next validated the induction of *S100A8*, *S100A9*, and *S100A12* (Figure 4B) mRNA expression in butyrate macrophages compared to control macrophages by qPCR at baseline. Butyrate-induced *S100A8* and *S100A9* expression is not only observed at baseline but also in LPS stimulated cells suggesting that a short term inflammatory stimulus does not override the butyrate induced antimicrobial function (Figures S5A and S5B).

Analysis of control and butyrate macrophage supernatants by ELISA confirmed the significant increase in calprotectin protein secretion by butyrate macrophages (Figure 4C). Similarly, intracellular staining of the S100A8 and S100A9 proteins by flow cytometry shows a striking upregulation of calprotectin in macrophages after 5 days of differentiation with butyrate (Figure 4D). Quantification of calprotectin protein in the lysate of control and butyrate macrophages at steady state by immunoblot also confirmed a significant increase in butyrate macrophages (Figures 4E and 4F).

Calprotectin is a well-studied antimicrobial protein that is capable of enhancing bacterial killing via different mechanisms such as sequestration of metal ions such as zinc (Zn^{2+}) and manganese (Mn^{2+}) (Hood and Skaar, 2012; Kehl-Fie et al., 2011). To confirm the functional role of *S100A8/9* in the elimination of invasive intracellular bacteria, we differentiated control and butyrate macrophages in the presence of siRNAs against *S100A8 and S100A9* before performing a gentamicin protection assay. The significant silencing of both S100A8 and S100A9 genes (Figures S5C and S5D) found in butyrate macrophages treated with *S100A8/S100A9* siRNA was associated with a significant increase of bacterial load compared to untreated or scrambled siRNA treated control and butyrate macrophages (Figure 4G). Together these results indicate that the presence of butyrate during monocyte to macrophage differentiation drives a synergistic program of antimicrobial LC3-associated host defense and production of antimicrobial peptides, which together promote cellular antimicrobial activity.

(C) Examples of the expression of marker genes identified (in both control and butyrate macrophages) for cells in each of the five clusters (Benjamini Hochberg [BH] adjusted p values < 0.05).

(D) Changes in gene expression between control and butyrate macrophages. The genes shown in the heatmap are significantly differentially expressed (BH adjusted p value < 0.05, $\log_2$ fold-change > 2) between control and butyrate macrophages in at least one of the four clusters of differentiated (non-cell-cycle associated) macrophages.

(E) Selected examples of significantly enriched Gene Ontology (GO) categories among genes up (group i) or down (groups ii and iii) regulated by butyrate (BH adjusted p value < 0.05, Fishers exact test). Please also see Figure S4 and Tables S1–S3.

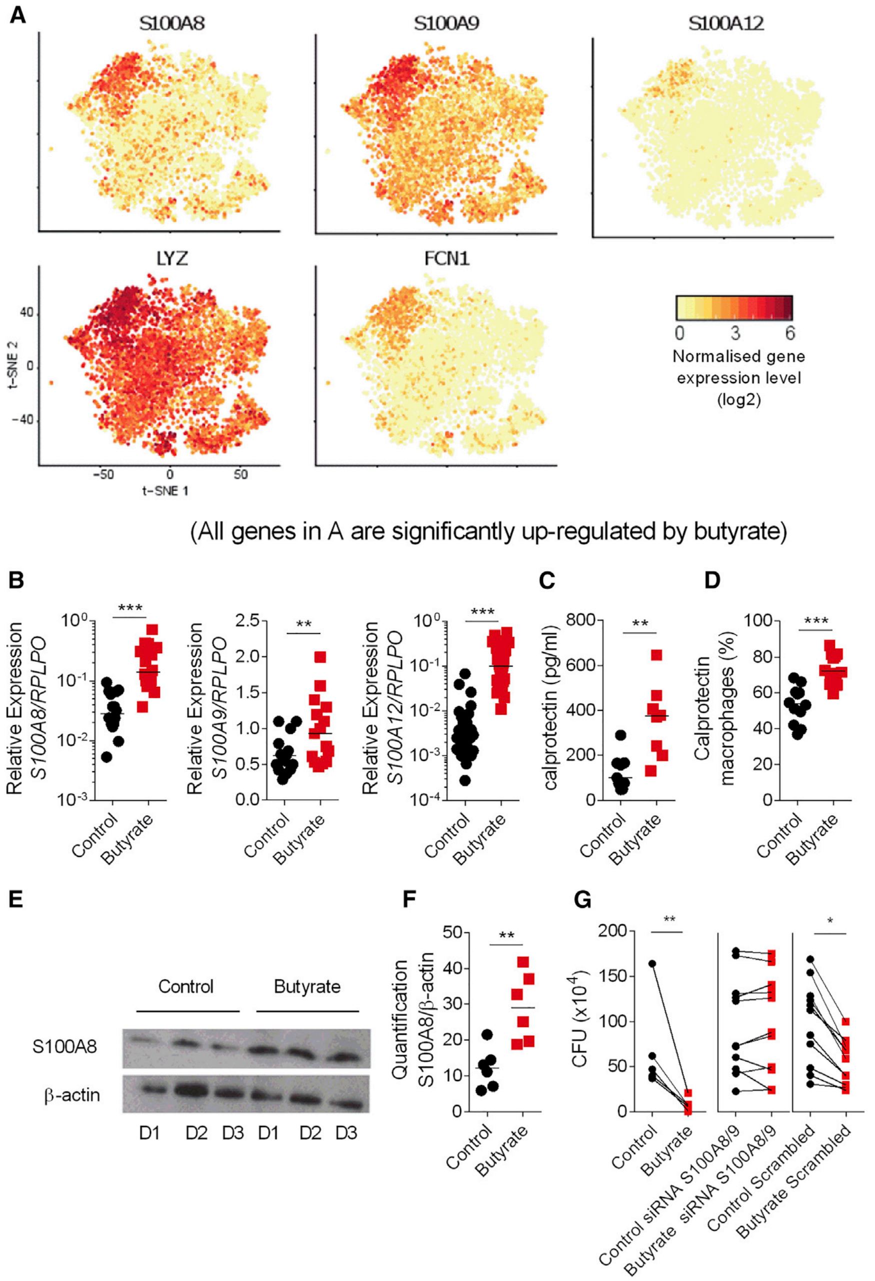

Figure 4. Upregulation of Calprotectin in Macrophages Differentiated in Presence of Butyrate

(A) t-SNE plots of significantly (BH adjusted p value < 0.05) butyrate-induced antimicrobial genes (*S100A8*, *S100A9*, *S100A12*, *LYZ*, and *FCN1*) at the single-cell level.

(B) Gene expression of *S100A8*, *S100A9*, *S100A12* at steady state in control and butyrate macrophages.

(C) Quantification of calprotectin in the supernatant by ELISA.

(D) Intra-cellular expression of *S100A8* and *S100A9* protein at steady state by flow cytometry.

(E) Representative immunoblot of calprotectin and β-actin at steady state from three donors.

(F) Quantification of calprotectin expression at steady state.

(G) Gentamicin protection assay against *Salmonella* performed on control and butyrate macrophages treated with scrambled siRNA or a mix of *S100A8* and *S100A9* siRNA.

Each dot represents one donor. Statistical significance was determined using Mann-Whitney U test *p < 0.05, **p < 0.01 and ***p < 0.001.

Please also see Figure S5.

(Rooks and Garrett, 2016). HDACs remove acetyl groups on specific lysine residues from histones and non-histone proteins regulating gene expression by modulating chromatin structure. We tested the ability of butyrate to inhibit HDAC activity in macrophages, using histone H3 and H4 acetylation as an indirect readout. Butyrate macrophages displayed a higher amount of acetylated H3 and H4 compared to controls (Figure 5E). Furthermore, butyrate macrophages showed a significant decrease of the tri-methylation of lysine 27 on histone 3 (3MeH3K27), associated with chromatin repression (Figure 5F), as well as increased acetylation of lysine 27 on histone 3 (AcH3K27), which is associated with more open chromatin (Figure 5G). In support of these findings, treatment of macrophages with pan-HDAC inhibitors such as valproate, phenyl-butyrate, and veronistat (SAHA) increased their antimicrobial activity (Figure 5H and 5J) and increased expression of *S100A8* mRNA (Figure 5I and 5K).

We next investigated the specificity of butyrate-mediated HDAC inhibition in macrophages. HDACs are divided into 2 main classes: class I and class II (subdivided into IIa and IIb) (Haberland et al., 2009). We initially treated macrophages with TMP195, an inhibitor of HDAC class IIa (Davie, 2003) that has been used to activate tumor-associated macrophages (Guerriero et al., 2017) and with tubacin that targets HDAC class IIb. However, neither macrophages differentiated in the presence of TMP195 nor tubacin showed changes in bactericidal function (Figure 5L) or upregulation of *S100A8* mRNA (Figure 5M)

Butyrate Promotes Antimicrobial Activity in Macrophages through Its HDAC Inhibitory Function

Since butyrate can signal via G protein–coupled receptors (GPCRs) (Blad et al., 2012), we studied the role of GPCRs in butyrate-induced bacterial clearance. We found that mRNA expression of the known butyrate receptors *FFAR2*, *FFAR3*, and *HACR2* genes were more highly expressed in myeloid populations than lymphoid and dendritic cell populations (Figures 5A–5C). However, macrophages differentiated in the presence of both butyrate and the GPCR inhibitor pertussis toxin (PT) still exhibited enhanced anti-bacterial activity (Figure 5D) suggesting that GPCRs are not required for butyrate induced antimicrobial functions.

We next tested the role of the histone deacetylase (HDAC) inhibitory function of butyrate in enhancing antimicrobial function

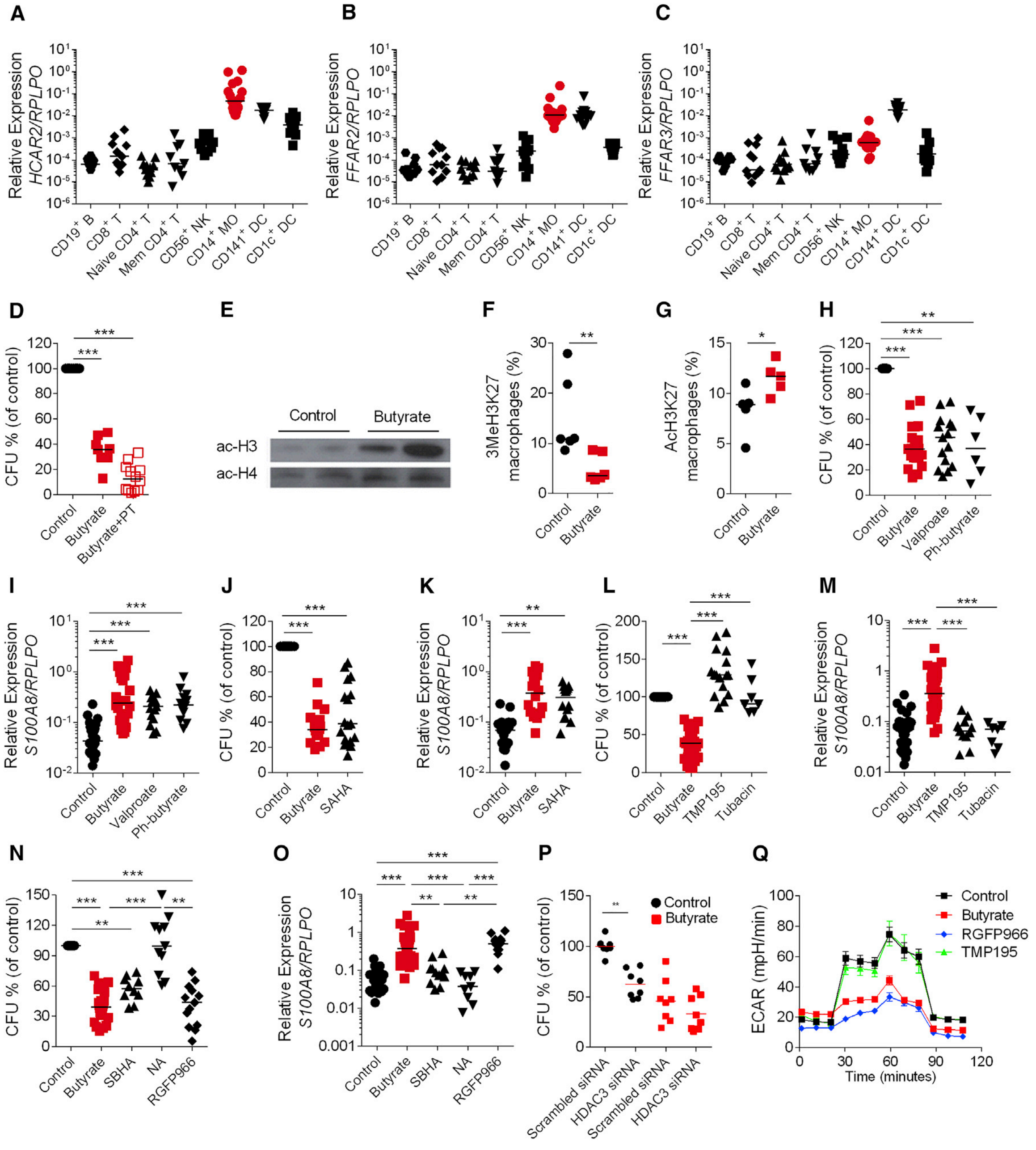

Figure 5. Butyrate Promotes Antimicrobial Activity in Macrophages via HDAC Inhibitory Function

(A–C) Gene expression of *hcar2* (A), *ffar2* (B), and *ffar3* (C) on flow cytometry sorted $CD19^+$ B, $CD8^+$ T, naive $CD4^+$ T, memory $CD4^+$ T, $CD56^+$ $CD3^-$ NK cells, $CD14^+$ monocytes, $CD141^+$ DCs, and $CD1c^+$ DCs from the blood of healthy donors. Each dot represents one donor.

(D) Gentamicin protection assay on control and butyrate macrophages, or butyrate macrophages differentiated in the presence of butyrate with pertussis toxin (PT).

(E) Protein expression of acetyled histone 3 (Ac-H3) and acetyled histone 4 (Ac-H4) in control and butyrate macrophages (data from 2 individual donors per condition).

(F and G) Intra-cellular expression of tri-methylated lysine27 on histone 3 (3MeH3K27) (F) and acetylated lysine 27 on histone 3 (AcH3K27) (G) protein on control and butyrate macrophages by flow cytometry.

(H, J, L, and N) Gentamicin protection assay on control macrophages, butyrate macrophages, and macrophages differentiated in the presence of valproate, phenylbutyrate (H), SAHA (J), TMP195, and tubacin (L), SBHA, 1-naphthohydroxamic acid (NA), or RGFP966 (N).

(I, K, M, and O) Gene expression of *S100A8* in control macrophages, butyrate macrophages, and macrophages differentiated in the presence of valproate, phenylbutyrate (I), SAHA (K), TMP195, and tubacin (M), SBHA, 1-naphthohydroxamic acid (NA), or RGFP966 (O).

(P) Gentamicin protection assay in control and butyrate macrophages with and without HDAC3 siRNA-mediated gene silencing.

(Q) Extracellular acidification rate in control and butyrate macrophages, as well as in RGFP966 and TMP195 treated macrophages. Data represent the mean of three biological replicates.

(legend continued on next page)

suggesting that butyrate does not act via inhibition of class II HDAC. Next we treated macrophages with several inhibitors that target class I HDACs: valproate (class I HDACi and reduces protein expression of HDAC2), SBHA (targeting HDAC1 and 3), 1-naphthohydroxamic Acid (NA; targeting HDAC8, 1, and 6), and RGFP966 (a HDAC3-specific inhibitor) (Jia et al., 2016). We found that macrophages differentiated with valproate and SBHA but not NA showed a significant reduction of CFU compared to control macrophages (Figure 5N). Similarly, the HDAC3 inhibitor RGFP966 induced elevated bacterial clearance (Figure 5N) and increased *S100A8* mRNA expression (Figure 5O). Silencing of HDAC3 with siRNA resulted in significantly increased *Salmonella* clearance at baseline in control macrophages. Importantly, butyrate failed to enhance bacterial killing in macrophages with HDAC3 silencing (Figure 5P and Figure S5E). Together these results show that inhibition of HDAC3 is sufficient to induce the differentiation of macrophages with bactericidal functions and that butyrate's anti-microbial effects on macrophages are dependent on HDAC3. Macrophages differentiated with the HDAC3 inhibitor RGFP966 but not TMP195 also showed reduced glycolysis similar to butyrate macrophages indicating inhibition of HDAC3 is upstream of both metabolic changes and anti-microbial responses in macrophages (Figure 5Q).

Butyrate Treatment Promotes Antibacterial Activity in Intestinal Macrophages and Restricts Bacterial Translocation *In Vivo*

To test whether the effects of butyrate on macrophage differentiation are relevant *in vivo*, we treated C57BL/6 mice with a daily oral dose of butyrate or water for 7 days. At day 7, we sorted colonic macrophages by flow cytometry and performed an *ex vivo* gentamicin protection assay. In line with our *in vitro* findings, colonic macrophages from butyrate treated-mice exhibited higher antimicrobial activity compared to controls (Figure 6A). To confirm that the increase in bacterial killing is specific to colonic macrophages, we induced the differentiation of bone marrow (BM) progenitors from butyrate-treated or from untreated mice in the presence of M-CSF to determine whether macrophages derived from the BM of butyrate-treated animals displayed increased antimicrobial activity. Macrophages differentiated from butyrate-treated mice did not show any improvement in antibacterial function compared to macrophages differentiated in the presence of butyrate (Figure 6B) indicating butyrate functions through local effects on intestinal macrophages.

We next investigated whether mice treated orally with butyrate showed a reduction in dissemination of bacteria to peripheral organs after infection. Mice were treated orally for 7 days with butyrate prior to oral infection with *Salmonella* or *C. rodentium*. Two days after *Salmonella* infection, bacterial dissemination was quantified. Butyrate-treated mice showed a significant reduction of bacterial dissemination in the mesenteric lymph node (MLN), spleen, and liver compared to untreated mice (Figure 6C), though *Salmonella* load in the caecal contents of both groups of mice was not different (Figure 6D). Salmonella infection was associated with a mild inflammatory response in the colon, which was reduced in butyrate treated mice (Figures 6E and 6F). This effect was not associated with an increase in the frequency of $CD4^+Foxp3^+$ regulatory T cells (Figure S6).

Similar results were observed after infection with *C. rodentium*. While both groups had similar weight curves during infection (Figure 6G), butyrate-treated mice displayed a marked reduction in the dissemination of *C. rodentium* to the liver and spleen (Figure 6H) indicating that butyrate promotes anti-microbial defense *in vivo*.

These results indicate that oral butyrate supplementation promotes the differentiation of intestinal macrophages that possess strong antimicrobial activity and that this reduces the dissemination of pathogenic bacteria.

DISCUSSION

The intestinal immune system is highly adapted to provide host defense in the face of pathogens while retaining a mutualistic response with commensal bacterial. Bacterial fermentation products SCFA are major mediators of host-microbe cross talk in the intestine, controlling the development and maintenance of indigenous bacterial communities on the one hand and differentiation and maturation of intestinal tissue and immune cells on the other (Rooks and Garrett, 2016). Here we have identified a role for butyrate as a differentiation factor for monocyte-derived macrophages that enhances cell-intrinsic antimicrobial functions. We have shown that butyrate acted via its HDAC3 inhibitory function to alter metabolism and induce production of anti-microbial peptides leading to enhanced bactericidal function *in vitro* and *in vivo*.

Butyrate caused a profound metabolic and immunologic alteration in macrophages that is in many aspects opposite to the well-known pro-inflammatory LPS stimulation or “trained immunity” seen in pro-inflammatory M1 macrophages. LPS treated macrophages show increased glycolysis, inhibition of AMPK, increase of mTOR signaling, as well as reduced carbohydrate kinase-like protein (CARKL) mRNA that flux into the pentose phosphate pathway (Kelly and O'Neill, 2015). By contrast, macrophages differentiated in the presence of butyrate show reduced glycolysis, higher amounts of AMP and increased AMPK phosphorylation at Thr172, a residue that is critical for enzyme activity (Hardie et al., 2012). Butyrate macrophages also show reduced S6 phosphorylation, a surrogate marker for inhibition of mTOR. Reductions in mTOR, which is a known positive regulator of glycolytic enzymes such as hexokinase II, glyceraldehyde 3-phosphate dehydrogenase, and lactate dehydrogenase-B (Sun et al., 2011) may explain the reduced glycolysis observed in butyrate macrophages. In addition, butyrate treated macrophages show increased amounts of ribulose 5-phosphate and reduced intracellular glucose (despite normal glucose uptake) suggesting an

For the gentamicin protection assay, the percentage of CFU (of control) was calculated from mean value for control group.
Each dot represents one independent donor. For pairwise comparison Mann-Whitney U test was performed and for multiple group comparisons a one-way ANOVA (Kruskal-Wallis test) was performed. *$p < 0.05$, **$p < 0.01$, and ***$p < 0.001$.
Please also see Figure S5E.

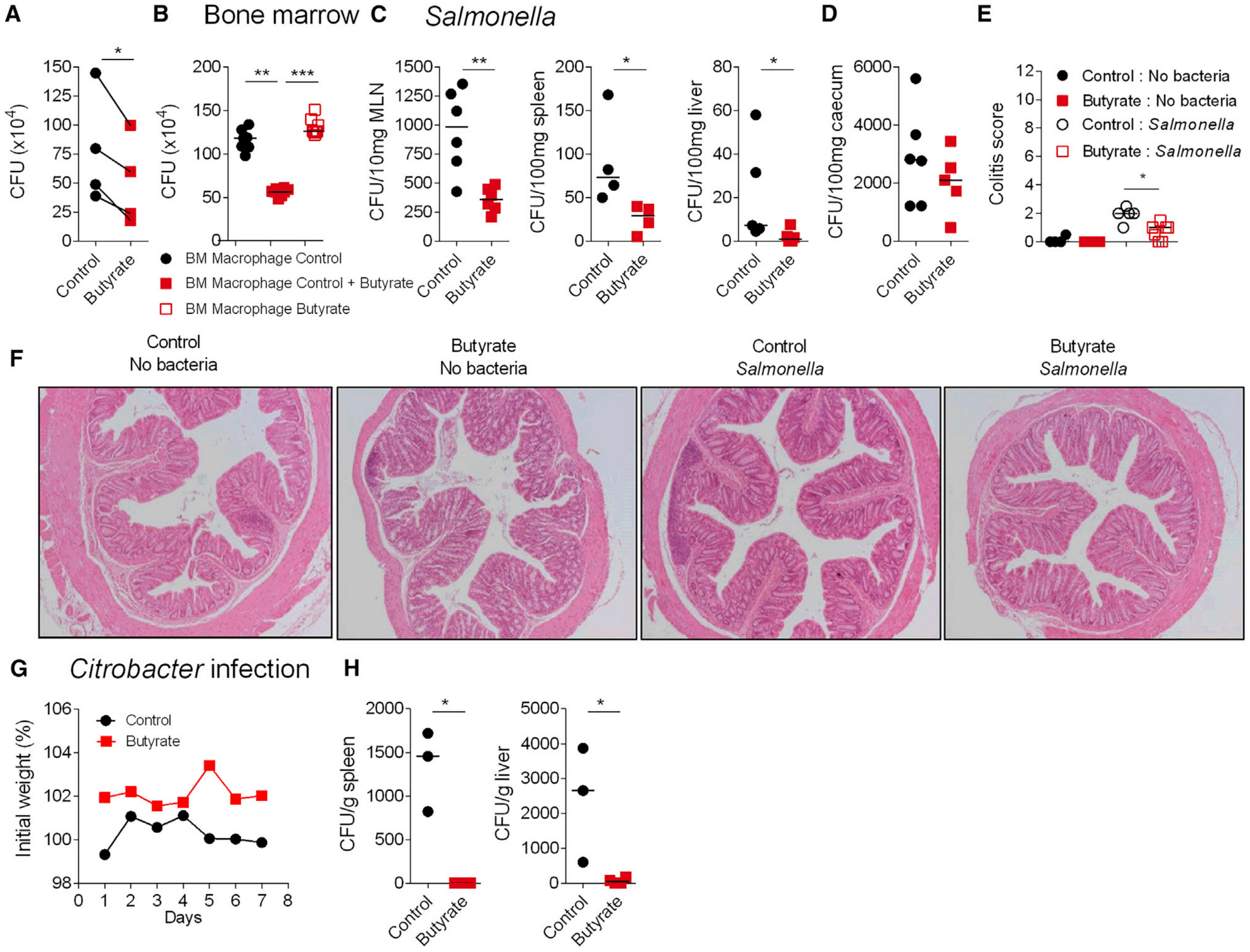

Figure 6. Induction of Antimicrobial Activity by Butyrate in Macrophages *In Vivo*

(A) WT mice received sodium butyrate in drinking water (150 mM final concentration) or PBS control for 7 days. At day 7, colonic segments were digested, macrophages were isolated by flow cytometry sorting and a gentamicin protection assay was performed. Each dot represents macrophages pooled from ten mice. Four independent experiments are shown.

(B) Mouse bone marrow progenitor cells from mice gavaged with sodium butyrate or with PBS were differentiated into macrophages in the presence of M-CSF or with M-CSF with butyrate as a positive control. A gentamycin assay was performed at day 7 of differentiation.

(C and D) Mice received butyrate or PBS 5 days prior to oral infection with *Salmonella typhymurium def aroA* (1×10^9 bacteria/mouse). 2 days post-infection bacterial dissemination was assessed in MLN, spleen, liver (C), and caecum (D). Each dot represents a mouse.

(E) Colitis score of control and butyrate-treated mice either uninfected or infected with *Salmonella*.

(F) Representative H&E stained colon sections from control and butyrate-treated mice either uninfected or infected with *Salmonella* (original magnification 100x).

(G and H) Mice were treated with 150 mM sodium butyrate or with PBS 3 days prior and every other day after oral infection with *Citrobacter rodentium* (1×10^9 bacteria/mouse). Mice were weighed daily. Lines represents mean of 3 mice (G). At day 7 post infection bacterial dissemination was assessed in the spleen and in the liver (H). Each dot represents a mouse. Statistical significance was determined using Mann-Whitney U test *$p < 0.05$, **$p < 0.01$, and ***$p < 0.001$.

Please also see Figure S6.

increased flux toward the pentose phosphate pathway. An increased flux toward the pentose phosphate pathway could contribute to the generation of NADPH, which may fuel the increased NADPH-oxidase-dependent ROS observed in butyrate macrophages both at baseline and also after *Salmonella* infection. Consistent with previous data that increased AMP kinase activity inhibits mTOR signaling and induces autophagy (Kelly and O'Neill, 2015; Shimobayashi and Hall, 2016), we found increased LC3 and increased bacterial clearance as a marker of effective autophagy in butyrate differentiated macrophages (Kim and Guan, 2015). Our results suggest an LC3-associated process since butyrate macrophages express high amounts of lipidated LC3-II protein detected by immunoblot, flow cytometry, and microscopy. However, we cannot differentiate whether the effects of butyrate are linked to canonical autophagy or non-canonical LC3-associated phagocytosis pathways (Martinez et al., 2015).

Single-cell RNA-sequencing identified five clusters of gene expression among human monocyte-derived macrophages with enrichment of distinct cellular processes reiterating the emerging concept of phenotypic and functional heterogeneity. Addition of butyrate increased expression of a number of anti-bacterial and

host defense genes supporting the marked increase in bacterial killing in butyrate macrophages at the population level. However, a butyrate-induced antimicrobial signature involving the expression of *S100A8*, *S100A9*, *S10012*, *LYZ*, and *FCN1* was particularly pronounced in a subset of differentiated macrophages (cluster 2). The modulation of metabolic regulation toward LC3-associated processes in conjunction with induction of multiple antimicrobial genes within those cells provides the machinery for intracellular bacterial segregation and effector mechanisms for killing. Calprotectin (a heterodimer of S100A8 and S100A9) mRNA and protein was detected in butyrate macrophages. Gene silencing of calprotectin ablated the increased anti-bacterial properties of butyrate macrophages, illustrating the functional importance of this antimicrobial peptide. Calprotectin can inhibit the growth of intracellular pathogens by a number of mechanisms including chelation of bivalent cations (Zaia et al., 2009), activation of NADPH oxidase (Kerkhoff et al., 2005), or induction of lipidated LC3-II (Wang et al., 2015).

We have shown that HDAC3 inhibition by butyrate (or the specific HDAC3i RGFP966) drove the differentiation of macrophages, altered their metabolism, and enhanced gene expression of antimicrobial peptides uncoupled from increased inflammatory cytokine production. Butyrate has been shown to act as an HDACi to inhibit acute LPS stimulated inflammatory cytokine production by murine macrophages *in vitro* (Chang et al., 2014). In that setting HDAC3 deficiency or inhibition has anti-inflammatory function (Chen et al., 2012; Leus et al., 2016). *Lyz2-cre*$^{+}$, *Hdac3*$^{f/f}$ macrophages also polarize more strongly toward an M2 phenotype when stimulated with IL-4 (Mullican et al., 2011). However, that function is context dependent and requires IL-4 signaling. Our findings complement and extend previous studies by showing that butyrate through its HDAC3i function can also induce the differentiation of a specialized macrophage anti-microbial state in the absence of changes in inflammatory cytokine production. Together these studies support a model in which butyrate imprints a non-inflammatory and antimicrobial program in macrophages that promotes intestinal homeostasis. This is consistent with the concept that butyrate-producing commensal bacteria shape host microbial crosstalk to promote a stable relationship, avoiding the disruptive effects of inflammation on the ecosystem. This is an important mechanism, since genetic defects in bacterial handling and reduction in butyrate producing bacteria have been linked to IBD (Baxt and Xavier, 2015; Cadwell, 2016; Frank et al., 2007). This also fits with the observation that human intestinal macrophages showed reduced inflammatory potential compared to peripheral blood monocytes (Bujko et al., 2018). Our results show that mice pre-treated with butyrate exhibit a reduction of systemic dissemination when orally infected with *C. rodentium* or *Salmonella*. Although we cannot exclude that in the *in vivo* setting butyrate promotes antimicrobial barrier function via several mechanisms, macrophages sorted from the colon of butyrate-fed mice display an increase in bactericidal function and an upregulation of *S100A8* mRNA suggesting that these cells contribute to antimicrobial homeostasis.

Epidemiological studies suggest that early life exposure to antibiotics is a risk factor and a high-fiber diet (associated with increased luminal butyrate concentrations) is a protective factor in IBD (Ananthakrishnan et al., 2018; Cushing et al., 2015). In a model system, broad spectrum antibiotics causes butyrate depletion and butyrate-responsive macrophage and T cell dysfunction (Scott et al., 2018). Restoring antimicrobial function via butyrate in intestinal macrophages may therefore be a universal mechanism to prevent or treat IBD. It is feasible to pharmacologically increase butyrate concentrations via enema *in vivo* in portal vein blood to 92.2 μmol/L (van der Beek et al., 2015). Although it is not yet clear whether enemas containing butyrate or a cocktail of SCFAs can ameliorate intestinal inflammation (Cushing et al., 2015), treatments that harness butyrate effector mechanisms might have the potential to prevent IBD or reduce relapse activity. Our findings suggest clear differences between the anti-tumor inflammatory macrophages induced by the class II HDACi TMP195 and antimicrobial macrophages induced by HDAC3 targeting inhibitors suggesting a concept of differential imprinting of macrophage function via selective HDAC inhibition. It remains to be determined whether selective HDAC3 inhibitors would have an advantage beyond their natural counterpart butyrate. Indeed, HDAC3 inhibition might induce complex effects on other cell types such as those present in the epithelium (Alenghat et al., 2013).

In conclusion, our results demonstrate that butyrate directs the differentiation of homeostatic macrophages that possess strong antimicrobial activity and play an important role in preventing the dissemination of bacteria beyond the intestinal barrier. Butyrate educates developing macrophages via HDAC3 inhibition by regulating their metabolic and transcriptional program. This has implications for prevention and therapy of disorders that are associated with intestinal inflammation, as well as systemic infection.

STAR★METHODS

Detailed methods are provided in the online version of this paper and include the following:

- KEY RESOURCES TABLE
- HUMAN MONOCYTES, DIFFERENTIATION AND CELL CULTURE
- REAGENTS
- BACTERIAL STRAINS AND GENTAMICIN PROTECTION ASSAY
- SEAHORSE ASSAY
- METABOLOMICS ANALYSIS BY LC-MS
  - Metabolite extraction from cells
  - LC-MS/MS analysis
  - Data processing
  - Data analysis
- AMPK ELISA
- ANNEXIN V AND 7AAD STAINING
- MEASUREMENT OF REACTIVE OXYGEN SPECIES
- CONFOCAL MICROSCOPY
- FLOW CYTOMETRY
- CD45^{+}CD3^{+}CD4^{+}FOXP3^{+} T CELLS QUANTIFICATION
- ANALYSIS OF SINGLE-CELL TRANSCRIPTOMICS DATA
- QUANTITATIVE PCR
- QUANTITATION OF CYTOKINE SECRETION
- TRANSFECTION OF PRIMARY HUMAN MACROPHAGES

- IMMUNOBLOT
- ISOLATION OF LYMPHOID AND MYELOID POPULATIONS FROM HUMAN BLOOD
- EXPERIMENTAL MODEL AND SUBJECT DETAILS
- SODIUM BUTYRATE TREATMENT AND INFECTION
- HISTOLOGY SCORE
- ISOLATION OF MOUSE COLONIC MACROPHAGES
- BONE MARROW DERIVED MACROPHAGES
- PHAGOCYTOSIS ASSAY
- STATISTICS
- DATA AND SOFTWARE AVAILABILITY

SUPPLEMENTAL INFORMATION

Supplemental Information includes six figures and three tables and can be found with this article online at https://doi.org/10.1016/j.immuni.2018.12.018.

ACKNOWLEDGMENTS

We thank all volunteers and blood donors for participation in this study. We thank Sebastian Rogatti for technical assistance, Helen Ferry and Jonathan Webber for flow cytometry cell sorting, and the staff of our animal houses. We acknowledge the contribution of the BRC Gastrointestinal biobank (11/YH/0020, 16/YH/0247), supported by the National Institute for Health Research (NIHR) Oxford Biomedical Research Centre (BRC). This work was supported by the Wellcome Trust UK (095688/Z/11/Z: F.P., D.G.W.J., and A.C.), the BBSRC (BB/I005609/1: FP, CVA-C, JS), the Leona M. and Harry B. Helmsley Charitable Trust (FP, HHU, JS, MC), the Kennedy Trust for Rheumatology Research (SNS, K.C.R-A. and N.E.I.), CRUK grant (OCRC-DPHIL13-FF: FF) and the Crohn's & Colitis Foundation of America (HHU, FP and SP). *Salmonella enterica* serovar Typhimurium (*S.* Typhimurium)-expressing GFP was kindly provided by D. Holden (Imperial College, University of London, UK) and *adherent-invasive E. coli* reference strain LF82 (AIEC) by A. Darfeuille-Michaud. The graphical abstract was prepared including a graphic element from Les Laboratoires Servier. The views expressed are those of the authors and not necessarily those of the NHS, the NIHR, or the Department of Health.

AUTHOR CONTRIBUTIONS

J.S., S.P., I.A., M.C., F.F., A.C., J.M., D.G.W.J. performed experiments and K.C.R.-A., N.I., S.N.S. and J.M. bioinformatics analyses. S.N.S. supervised the single-cell analysis; C.V.A.-C. supervised initial experiments; F.P. and H.H.U. supervised the study. All authors discussed the data and manuscript.

DECLARATION OF INTEREST

None of the authors has a conflict of interest related to this article. H.H.U. received research support or consultancy fees from UCB Pharma, Eli Lilly, Boehringer Ingelheim, Pfizer, Celgene, and AbbVie. F.P. has received research support or consultancy fees from GSK, UCB Pharma, Medimmune, Janssen, Roche, and Eli Lilly.

Received: March 12, 2018
Revised: November 5, 2018
Accepted: December 14, 2018
Published: January 22, 2019

REFERENCES

Alenghat, T., Osborne, L.C., Saenz, S.A., Kobuley, D., Ziegler, C.G., Mullican, S.E., Choi, I., Grunberg, S., Sinha, R., Wynosky-Dolfi, M., et al. (2013). Histone deacetylase 3 coordinates commensal-bacteria-dependent intestinal homeostasis. Nature *504*, ,153–157.

Ananthakrishnan, A.N., Bernstein, C.N., Iliopoulos, D., Macpherson, A., Neurath, M.F., Ali, R.A.R., Vavricka, S.R., and Fiocchi, C. (2018). Environmental triggers in IBD: a review of progress and evidence. Nat. Rev. Gastroenterol. Hepatol. *15*, 39–49.

Arnold, I.C., Mathisen, S., Schulthess, J., Danne, C., Hegazy, A.N., and Powrie, F. (2016). CD11c(+) monocyte/macrophages promote chronic Helicobacter hepaticus-induced intestinal inflammation through the production of IL-23. Mucosal Immunol. *9*, 352–363.

Arpaia, N., Campbell, C., Fan, X., Dikiy, S., van der Veeken, J., deRoos, P., Liu, H., Cross, J.R., Pfeffer, K., Coffer, P.J., and Rudensky, A.Y. (2013). Metabolites produced by commensal bacteria promote peripheral regulatory T-cell generation. Nature *504*, 451–455.

Ashburner, M., Ball, C.A., Blake, J.A., Botstein, D., Butler, H., Cherry, J.M., Davis, A.P., Dolinski, K., Dwight, S.S., Eppig, J.T., et al.; The Gene Ontology Consortium (2000). Gene ontology: tool for the unification of biology. Nat. Genet. *25*, 25–29.

Bain, C.C., Bravo-Blas, A., Scott, C.L., Perdiguero, E.G., Geissmann, F., Henri, S., Malissen, B., Osborne, L.C., Artis, D., and Mowat, A.M. (2014). Constant replenishment from circulating monocytes maintains the macrophage pool in the intestine of adult mice. Nat. Immunol. *15*, 929–937.

Baxt, L.A., and Xavier, R.J. (2015). Role of Autophagy in the Maintenance of Intestinal Homeostasis. Gastroenterology *149*, 553–562.

Blad, C.C., Tang, C., and Offermanns, S. (2012). G protein-coupled receptors for energy metabolites as new therapeutic targets. Nat. Rev. Drug Discov. *11*, 603–619.

Bujko, A., Atlasy, N., Landsverk, O.J.B., Richter, L., Yaqub, S., Horneland, R., Øyen, O., Aandahl, E.M., Aabakken, L., Stunnenberg, H.G., et al. (2018). Transcriptional and functional profiling defines human small intestinal macrophage subsets. J. Exp. Med. *215*, 441–458.

Butler, A., and Satija, R. (2018). Integrated analysis of single cell transcriptomic data across conditions, technologies, and species. Nat. Biotechnol. *36*, 411–420.

Cadwell, K. (2016). Crosstalk between autophagy and inflammatory signalling pathways: balancing defence and homeostasis. Nat. Rev. Immunol. *16*, 661–675.

Chang, P.V., Hao, L., Offermanns, S., and Medzhitov, R. (2014). The microbial metabolite butyrate regulates intestinal macrophage function via histone deacetylase inhibition. Proc. Natl. Acad. Sci. USA *111*, 2247–2252.

Chen, X., Barozzi, I., Termanini, A., Prosperini, E., Recchiuti, A., Dalli, J., Mietton, F., Matteoli, G., Hiebert, S., and Natoli, G. (2012). Requirement for the histone deacetylase Hdac3 for the inflammatory gene expression program in macrophages. Proc. Natl. Acad. Sci. USA *109*, E2865–E2874.

Choi, Y.J., Park, Y.J., Park, J.Y., Jeong, H.O., Kim, D.H., Ha, Y.M., Kim, J.M., Song, Y.M., Heo, H.S., Yu, B.P., et al. (2012). Inhibitory effect of mTOR activator MHY1485 on autophagy: suppression of lysosomal fusion. PLoS ONE *7*, e43418.

Cushing, K., Alvarado, D.M., and Ciorba, M.A. (2015). Butyrate and Mucosal Inflammation: New Scientific Evidence Supports Clinical Observation. Clin Transl Gastroen *6*.

Davie, J.R. (2003). Inhibition of histone deacetylase activity by butyrate. J. Nutr. *133* (7, Suppl), 2485S–2493S.

Finak, G., McDavid, A., Yajima, M., Deng, J., Gersuk, V., Shalek, A.K., Slichter, C.K., Miller, H.W., McElrath, M.J., Prlic, M., et al. (2015). MAST: a flexible statistical framework for assessing transcriptional changes and characterizing heterogeneity in single-cell RNA sequencing data. Genome Biol. *16*, 278.

Frank, D.N., St Amand, A.L., Feldman, R.A., Boedeker, E.C., Harpaz, N., and Pace, N.R. (2007). Molecular-phylogenetic characterization of microbial community imbalances in human inflammatory bowel diseases. Proc. Natl. Acad. Sci. USA *104*, 13780–13785.

French, K.E., Harvey, J., and McCullagh, J.S.O. (2018). Targeted and Untargeted Metabolic Profiling of Wild Grassland Plants identifies Antibiotic and Anthelmintic Compounds Targeting Pathogen Physiology, Metabolism and Reproduction. Sci. Rep. *8*, 1695.

Furusawa, Y., Obata, Y., Fukuda, S., Endo, T.A., Nakato, G., Takahashi, D., Nakanishi, Y., Uetake, C., Kato, K., Kato, T., et al. (2013). Commensal microbe-derived butyrate induces the differentiation of colonic regulatory T cells. Nature *504*, 446–450.

Guerriero, J.L., Sotayo, A., Ponichtera, H.E., Castrillon, J.A., Pourzia, A.L., Schad, S., Johnson, S.F., Carrasco, R.D., Lazo, S., Bronson, R.T., et al. (2017). Class IIa HDAC inhibition reduces breast tumours and metastases through anti-tumour macrophages. Nature *543*, 428–432.

Haberland, M., Montgomery, R.L., and Olson, E.N. (2009). The many roles of histone deacetylases in development and physiology: implications for disease and therapy. Nat. Rev. Genet. *10*, 32–42.

Hardie, D.G., Ross, F.A., and Hawley, S.A. (2012). AMPK: a nutrient and energy sensor that maintains energy homeostasis. Nat. Rev. Mol. Cell Biol. *13*, 251–262.

Hood, M.I., and Skaar, E.P. (2012). Nutritional immunity: transition metals at the pathogen-host interface. Nat. Rev. Microbiol. *10*, 525–537.

Huang, J., Canadien, V., Lam, G.Y., Steinberg, B.E., Dinauer, M.C., Magalhaes, M.A.O., Glogauer, M., Grinstein, S., and Brumell, J.H. (2009). Activation of antibacterial autophagy by NADPH oxidases. Proc. Natl. Acad. Sci. USA *106*, 6226–6231.

Inoki, K., Zhu, T., and Guan, K.L. (2003). TSC2 mediates cellular energy response to control cell growth and survival. Cell *115*, 577–590.

Izcue, A., Hue, S., Buonocore, S., Arancibia-Cárcamo, C.V., Ahern, P.P., Iwakura, Y., Maloy, K.J., and Powrie, F. (2008). Interleukin-23 restrains regulatory T cell activity to drive T cell-dependent colitis. Immunity *28*, 559–570.

Jia, H., Wang, Y., Morris, C.D., Jacques, V., Gottesfeld, J.M., Rusche, J.R., and Thomas, E.A. (2016). The Effects of Pharmacological Inhibition of Histone Deacetylase 3 (HDAC3) in Huntington's Disease Mice. PLoS ONE *11*, e0152498.

Kaiko, G.E., Ryu, S.H., Koues, O.I., Collins, P.L., Solnica-Krezel, L., Pearce, E.J., Pearce, E.L., Oltz, E.M., and Stappenbeck, T.S. (2016). The Colonic Crypt Protects Stem Cells from Microbiota-Derived Metabolites. Cell *165*, 1708–1720.

Kanehisa, M., and Goto, S. (2000). KEGG: kyoto encyclopedia of genes and genomes. Nucleic Acids Res. *28*, 27–30.

Kehl-Fie, T.E., Chitayat, S., Hood, M.I., Damo, S., Restrepo, N., Garcia, C., Munro, K.A., Chazin, W.J., and Skaar, E.P. (2011). Nutrient metal sequestration by calprotectin inhibits bacterial superoxide defense, enhancing neutrophil killing of Staphylococcus aureus. Cell Host Microbe *10*, 158–164.

Kelly, B., and O'Neill, L.A.J. (2015). Metabolic reprogramming in macrophages and dendritic cells in innate immunity. Cell Res. *25*, 771–784.

Kerkhoff, C., Nacken, W., Benedyk, M., Dagher, M.C., Sopalla, C., and Doussiere, J. (2005). The arachidonic acid-binding protein S100A8/A9 promotes NADPH oxidase activation by interaction with p67phox and Rac-2. FASEB J. *19*, 467–469.

Kim, Y.C., and Guan, K.L. (2015). mTOR: a pharmacologic target for autophagy regulation. J. Clin. Invest. *125*, 25–32.

Leus, N.G., van der Wouden, P.E., van den Bosch, T., Hooghiemstra, W.T., Ourailidou, M.E., Kistemaker, L.E., Bischoff, R., Gosens, R., Haisma, H.J., and Dekker, F.J. (2016). HDAC 3-selective inhibitor RGFP966 demonstrates anti-inflammatory properties in RAW 264.7 macrophages and mouse precision-cut lung slices by attenuating NF-κB p65 transcriptional activity. Biochem. Pharmacol. *108*, 58–74.

Liberzon, A., Subramanian, A., Pinchback, R., Thorvaldsdóttir, H., Tamayo, P., and Mesirov, J.P. (2011). Molecular signatures database (MSigDB) 3.0. Bioinformatics *27*, 1739–1740.

Love, M.I., Huber, W., and Anders, S. (2014). Moderated estimation of fold change and dispersion for RNA-seq data with DESeq2. Genome Biol. *15*, 550.

Martinez, J., Malireddi, R.K.S., Lu, Q., Cunha, L.D., Pelletier, S., Gingras, S., Orchard, R., Guan, J.-L., Tan, H., Peng, J., et al. (2015). Molecular characterization of LC3-associated phagocytosis reveals distinct roles for Rubicon, NOX2 and autophagy proteins. Nat. Cell Biol. *17*, 893–906.

Mullican, S.E., Gaddis, C.A., Alenghat, T., Nair, M.G., Giacomin, P.R., Everett, L.J., Feng, D., Steger, D.J., Schug, J., Artis, D., and Lazar, M.A. (2011). Histone deacetylase 3 is an epigenomic brake in macrophage alternative activation. Genes Dev. *25*, 2480–2488.

Peloquin, J.M., Goel, G., Villablanca, E.J., and Xavier, R.J. (2016). Mechanisms of Pediatric Inflammatory Bowel Disease. Annu. Rev. Immunol. *34*, 31–64.

Riffelmacher, T., Clarke, A., Richter, F.C., Stranks, A., Pandey, S., Danielli, S., Hublitz, P., Yu, Z., Johnson, E., Schwerd, T., et al. (2017). Autophagy-Dependent Generation of Free Fatty Acids Is Critical for Normal Neutrophil Differentiation. Immunity *47*, 466–480 e465.

Rooks, M.G., and Garrett, W.S. (2016). Gut microbiota, metabolites and host immunity. Nat. Rev. Immunol. *16*, 341–352.

Scott, N.A., Andrusaite, A., Andersen, P., Lawson, M., Alcon-Giner, C., Leclaire, C., Caim, S., Le Gall, G., Shaw, T., Connolly, J.P.R., et al. (2018). Antibiotics induce sustained dysregulation of intestinal T cell immunity by perturbing macrophage homeostasis. Sci. Transl. Med. *10*, https://doi.org/10.1126/scitranslmed.aao4755.

Shimobayashi, M., and Hall, M.N. (2016). Multiple amino acid sensing inputs to mTORC1. Cell Res. *26*, 7–20.

Sinha, R., Stanley, G., Gulati, G.S., Ezran, C., Travaglini, K.J., Wei, E., Chan, C.K.F., Nabhan, A.N., Su, T., Morganti, R.M., et al. (2017). Index Switching Causes "Spreading-Of-Signal" Among Multiplexed Samples In Illumina HiSeq 4000 DNA Sequencing. bioRxiv.

Smith, P.M., Howitt, M.R., Panikov, N., Michaud, M., Gallini, C.A., Bohlooly-Y, M., Glickman, J.N., and Garrett, W.S. (2013). The microbial metabolites, short-chain fatty acids, regulate colonic Treg cell homeostasis. Science *341*, 569–573.

Smythies, L.E., Sellers, M., Clements, R.H., Mosteller-Barnum, M., Meng, G., Benjamin, W.H., Orenstein, J.M., and Smith, P.D. (2005). Human intestinal macrophages display profound inflammatory anergy despite avid phagocytic and bacteriocidal activity. J. Clin. Invest. *115*, 66–75.

Subramanian, A., Tamayo, P., Mootha, V.K., Mukherjee, S., Ebert, B.L., Gillette, M.A., Paulovich, A., Pomeroy, S.L., Golub, T.R., Lander, E.S., and Mesirov, J.P. (2005). Gene set enrichment analysis: a knowledge-based approach for interpreting genome-wide expression profiles. Proc. Natl. Acad. Sci. USA *102*, 15545–15550.

Sun, Q., Chen, X., Ma, J., Peng, H., Wang, F., Zha, X., Wang, Y., Jing, Y., Yang, H., Chen, R., et al. (2011). Mammalian target of rapamycin up-regulation of pyruvate kinase isoenzyme type M2 is critical for aerobic glycolysis and tumor growth. Proc. Natl. Acad. Sci. USA *108*, 4129–4134.

Uhlig, H.H., and Powrie, F. (2018). Translating Immunology into Therapeutic Concepts for Inflammatory Bowel Disease. Annu. Rev. Immunol. *36*, 755–781.

Usami, M., Kishimoto, K., Ohata, A., Miyoshi, M., Aoyama, M., Fueda, Y., and Kotani, J. (2008). Butyrate and trichostatin A attenuate nuclear factor kappaB activation and tumor necrosis factor alpha secretion and increase prostaglandin E2 secretion in human peripheral blood mononuclear cells. Nutr. Res. *28*, 321–328.

van der Beek, C.M., Bloemen, J.G., van den Broek, M.A., Lenaerts, K., Venema, K., Buurman, W.A., and Dejong, C.H. (2015). Hepatic Uptake of Rectally Administered Butyrate Prevents an Increase in Systemic Butyrate Concentrations in Humans. J. Nutr. *145*, 2019–2024.

Van der Maaten, L., and Hinton, G. (2008). Visualizing data using t-SNE. J. Mach. Learn. Res. *9*, 2579–2605.

Varol, C., Vallon-Eberhard, A., Elinav, E., Aychek, T., Shapira, Y., Luche, H., Fehling, H.J., Hardt, W.-D., Shakhar, G., and Jung, S. (2009). Intestinal lamina propria dendritic cell subsets have different origin and functions. Immunity *31*, 502–512.

Villani, A.C., Satija, R., Reynolds, G., Sarkizova, S., Shekhar, K., Fletcher, J., Griesbeck, M., Butler, A., Zheng, S., Lazo, S., et al. (2017). Single-cell RNA-seq reveals new types of human blood dendritic cells, monocytes, and progenitors. Science *356*, https://doi.org/10.1126/science.aah4573.

Vinolo, M.A.R., Rodrigues, H.G., Hatanaka, E., Sato, F.T., Sampaio, S.C., and Curi, R. (2011a). Suppressive effect of short-chain fatty acids on

production of proinflammatory mediators by neutrophils. J. Nutr. Biochem. *22*, 849–855.

Vinolo, M.A.R., Rodrigues, H.G., Nachbar, R.T., and Curi, R. (2011b). Regulation of inflammation by short chain fatty acids. Nutrients *3*, 858–876.

Wang, T., Cai, G., Qiu, Y., Fei, N., Zhang, M., Pang, X., Jia, W., Cai, S., and Zhao, L. (2012). Structural segregation of gut microbiota between colorectal cancer patients and healthy volunteers. ISME J. *6*, 320–329.

Wang, J., Huang, C., Wu, M., Zhong, Q., Yang, K., Li, M., Zhan, X., Wen, J., Zhou, L., and Huang, X. (2015). MRP8/14 induces autophagy to eliminate intracellular Mycobacterium bovis BCG. J. Infect. *70*, 415–426.

Zaia, A.A., Sappington, K.J., Nisapakultorn, K., Chazin, W.J., Dietrich, E.A., Ross, K.F., and Herzberg, M.C. (2009). Subversion of antimicrobial calprotectin (S100A8/S100A9 complex) in the cytoplasm of TR146 epithelial cells after invasion by Listeria monocytogenes. Mucosal Immunol. *2*, 43–53.

STAR★METHODS

KEY RESOURCES TABLE

REAGENT or RESOURCE	SOURCE	IDENTIFIER
Antibodies		
Anti-CD45RO-APC Cy7/biotin (Clone UCHL1)	BioLegened	Catalog# 304228; RRID:AB_10895897
CD45RA PE Cy7/biotin (Clone HI100)	BioLegened	Catalog# 304125; RRID:AB_10709440
CD8-V500/biotin (Clone RPA-T8)	Biosciences	Catalog# 560775; RRID:AB_1937333
CD19- BV650/biotin (Clone HIB19)	BioLegend	Catalog# 302238; RRID:AB_2562097
7AAD	BioLegend	420403
Annexin V-BV510	BioLegend	640937
Anti-GLUT1 (Clone FAB1418P)	RnD Systems	Catalog# FAB1418P; RRID:AB_2191040
Anti-CD14 (Clone M5E2)	BioLegend	Catalog# 301804; RRID:AB_314186
Anti-CD11c (Clone B-Ly6)	BD Biosciences	Catalog# 657713; RRID:AB_2760137
Anti-HLA-DR (Clone G46-6)	BD Biosciences	Catalog# 561224; RRID:AB_10563765
Anti-Foxp3-PE-eFluor610 (Clone FJK-16s)	eBioscience	Catalog# 61-5773-80; RRID:AB_2574623
Anti-CD4-BV785 (Clone RM4-5)	BioLegend	Catalog# 100552; RRID:AB_2563053
Anti-CD45-BV650 (Clone 30-F11)	BioLegend	Catalog# 103151; RRID:AB_2565884
Anti-CD3-PECy7 (Clone 145-2C11)	BioLegend	Catalog# 100320; RRID:AB_312685
fixable viability dye	eBioscience	Catalog# 65-0865-14
Anti-S100A8 (Clone CF-145)	eBioscience	Catalog# 50-9745-42; RRID:AB_2574354
Anti-S100A9 (Clone MRP-14)	BioLegend	Catalog# 350706; RRID:AB_2564008
Anti-phosphor-S6 (ser235, ser236) (Clone cupk43k)	eBioscience	Catalog# 12-9007-42; RRID:AB_2572667
Anti-acetyl-histone H3 (lys27) (Clone D5E4)	New England biolabs	Catalog# 8173; RRID:AB_10949503
Anti-tri-methyl-histone H3 (lys27) (Clone C36B11)	New England biolabs	Catalog# 9733; RRID:AB_2616029
Anti-LC3B (Clone D11)	Cell signaling	Catalog# 3868; RRID:AB_2137707
Anti-β-actin (Clone 13E5)	Cell signaling	Catalog# 5125; RRID:AB_1903890
Anti-phosphor-S6 ribosomal protein (Ser235/236) (Clone D57.2.2E)	Cell signaling	Catalog# 4858; RRID:AB_916156
Anti-S100A8 (Clone EPR3554)	Abcam	Catalog# ab92331; RRID:AB_2050283
Anti-acetylated-H3 (Clone ab47915)	Abcam	Catalog# ab47915; RRID:AB_873860
Anti-acetylated-H4 (Clone EPR16606)	Abcam	Catalog# ab177790; RRID:AB_2732882
Anti-P62 lck (Clone 2/P62 LCK Ligand)	BD bioscience	Catalog# 610833; RRID:AB_398152
HRP-conjugated secondary antibody (anti-rabbit)	Cell signaling	Catalog# 7074S
HRP-conjugated secondary antibody (anti-rabbit)	Cell signaling	Catalog# 7076S
Bacterial strains		
Salmonella enterica serovar Typhimurium	D. Holden (Imperial College, University of London, UK)	NCTC 12023
CD-associated adherent invasive *Escherichia coli* (*AIEC*)	Arlette Darfeuille-Michaud lab France	LF82.30
Staphylococcus aureus	National collection of type cultures	NCTC 6571
Citrobacter. rodentium	Gad Frankel in Imperial College London	ICC169
Salmonella (mouse)	Gordon Dougan lab, Cambridge university	SL1344
Chemicals		
Human M-CSF	Preprotech	300-25-100
Sodium Butyrate	Sigma-Aldrich	303410-100G
Sodium Acetate	Sigma-Aldrich	S2889-250G
Sodium Propionate	Sigma-Aldrich	P1880

(*Continued on next page*)

Continued

REAGENT or RESOURCE	SOURCE	IDENTIFIER
Sodium Valproate	Sigma-Aldrich	S0930000
Sodium Phenylbutyrate	Sigma-Aldrich	SML0309
RGFP966	Sigma-Aldrich	SML1652
SAHA	Sigma-Aldrich	SML0061
SBHA	Sigma-Aldrich	390585
Tubacin	Sigma-Aldrich	SML0065
1-naphthohydroxamic	Sigma-Aldrich	SML0078
3-MA	Sigma-Aldrich	M9281-100MG
MHY1485	Sigma-Aldrich	SML0810
TMP195	Cellagen Technology	C8619-2
Gentamicin	Sigma-Aldrich	G1397-10ML
Seahorse XFe96 FluxPak	Agilent	102601-100
Seahorse XF base medium, sterile, 1 L, 2/pk	Agilent	102353-100
Feotal calf serum	Sigma-aldrich	F2442-6X500ML
Penicillin/streptomycin	Sigma-aldrich	P4333-100ML
HEPES	Sigma-aldrich	83264-100ML-F
Triton	Sigma-aldrich	T-8787-100ML
2NBDG	Sigma-aldrich	72987-1MG
Commercial assays		
Phagocytosis kit (Escherichia coli (K-12 strain) BioParticles™, Alexa Fluor™ 488 conjugate)	Thermofisher	E-13231 and CD14 MicroBeads, human; Source Miltenyi Biotec 130-050-201
Seahorse XF glycolysis stress test kit – 96x wells	Agilent	103020-100
Seahorse XF mito stress test kit – 96x wells	Agilent	103015-100
AMPK alpha-1,2 (Phospho) [pT172] Human ELISA Kit	Thermofisher	KHO0651
Softwares		
Cell Ranger (Version 2.0.2)	10X Genomics	https://support.10xgenomics.com
R (Version 3.4.2)	The Comprehensive R Archive Network (CRAN)	https://cran.r-project.org/
RStudio server (Version 1.1.383)	RStudio, Inc.	https://www.rstudio.com/
Pipelines for analyzing data generated with the 10x Genomics platform	Sansom Lab	https://github.com/sansomlab/tenx
Seurat R package (Version 2.1)	CRAN	https://CRAN.R-project.org/package=Seurat
DESeq2 (version 1.18.1)	Bioconductor	http://bioconductor.org/packages/DESeq2/
FlowJo	FlowJo	https://www.flowjo.com/
ImageJ	ImageJ	https://imagej.nih.gov/ij/
Prism	Prism - graphpad.com	https://www.graphpad.com/scientific-software/prism/
Others		
Seahorse XFe96 FluxPak	Agilent	102601-100
Deposited data		
Single cell data	Gene Expression Omnibus (GEO)	(GSE111049)

HUMAN MONOCYTES, DIFFERENTIATION AND CELL CULTURE

Human monocytes were isolated from leukocyte cones of healthy blood donors. Peripheral blood mononuclear cells (PBMC) were obtained by ficoll gradient. Monocyte-derived macrophages were generated using adherence method selection and M-CSF differentiation. Whole PBMC (50x10^6) were plated in RPMI-1640 medium for 90 min. After 2 washes with PBS, adherent monocytes were differentiated into macrophages over a 5 day period in presence of 100 ng/mL M-CSF in RPMI supplemented with 10% fetal calf serum (FCS) (Sigma-Aldrich), 100 U/mL penicillin, 100 μg/mL streptomycin, 30 mM HEPES, and 0.05 mM β-mercaptoethanol.

REAGENTS

All chemicals Butyrate (1mM), Acetate (1mM), Propionate (1mM), SAHA (1μM), SBHA (20μM), TMP195 (10 μM), RGFP966 (20 μM), 1-NA (10 μM), tubacin (1μM), 1-naphthohydroxamic acid, bafilomycin A1, 3-MA, MHY1485) were purchased from Sigma-Aldrich, unless specified otherwise. TMP195 was acquired from Cellagen technology and Cayman chemical respectively. Recombinant human and murine M-CSF were purchased from PeproTech.

BACTERIAL STRAINS AND GENTAMICIN PROTECTION ASSAY

Gentamicin protection assay was performed with the following strains: *Salmonella enterica* serovar Typhimurium (*Salmonella*)-expressing green-fluorescent protein (GFP) (NCTC 12023), CD-associated adherent invasive *Escherichia coli* (*AIEC*) reference strain LF82.30, *Staphylococcus aureus* (NCTC 6571). All strains were used at an MOI of 10 unless specified otherwise. For the gentamicin protection assay, macrophages were infected for 1h with *Salmonella* or *AIEC* or *S. aureus* or *C. rodentium* followed by gentamicin treatment for 2h. Cells were then lysed in 1% triton buffer and the lysate was plated on agar plates. Results are presented as absolute CFU count or % of mean control.

SEAHORSE ASSAY

Extra cellular acidification rate (ECAR) of the control and butyrate treated Macrophages was quantified by using a XF 96 extracellular flux analyzer (Seahorse Bioscience). 100,000 macrophages / well was plated in Seahorse base media was supplemented with 1% FCS, 1mM glutamine and 2mM sodium pyruvate. Plate were incubated in a Co_2 free incubator at 37°C for 1 h and later transferred to Seahorse machine for ECAR quantification. The assay was performed on 8 donors (biological replicates) with 5-8 technical replicates per donor. Similarly, for the mito-stress test, oxygen consumption rate (OCR) was quantified using a XF 96 extracellular flux analyzer (Seahorse Bioscience) as per the manufactures protocol. Base media for mito-stress was supplemented with 1% FCS, 1mM glutamine, 2mM sodium pyruvate and 10mM glucose.

METABOLOMICS ANALYSIS BY LC-MS

Metabolite extraction from cells

Metabolites were extracted from approximately $1x10^6$ cells (grown in cell culture dishes) by addition of 500 μL of ice cold 80% aqueous methanol. The supernatants were combined and filtered using a 3 kD ultrafilter (Millipore), dried in a SpeedVac and subsequently stored at −80°C. On the day of analysis, the dried extracts were re-constituted in 60 μL of ice cold 80% aqueous methanol. A quality control (QC) sample was made by combining 5 μL of each sample. This was injected at the start of the sequence and subsequently every 10 samples throughout the LC-MS/MS analyses.

LC-MS/MS analysis

Each sample was analyzed using two different LC-MS/MS methods utilizing two separate chromatographic systems. The first method used ion-chromatography coupled directly to Q-exactive HF Hybrid Quadrupole-Orbitrap mass spectrometer (IC-MS) (Thermo Scientific San Jose, CA). The second method utilized reversed-phase ultra-high performance chromatography (UHPLC) coupled directly to the same Q-exactive HF Hybrid Quadrupole-Orbitrap mass spectrometer (Thermo Ultimate 3000, Thermo Scientific, San Jose, CA). Both methods have been published previously and further details can be found in (French et al., 2018; Riffelmacher et al., 2017).

Data processing

Raw data files were processed using ProgenesisQI (Waters, Elstree, UK). This involved alignment of retention times, peak picking by identification of the presence of natural abundance isotope peaks, characterizing multiple adduct forms and identification of metabolites using our in-house database of authentic standards. Retention times, accurate mass values, relative isotope abundances and fragmentation patterns were compared between authentic standards and the samples measured. Identifications were accepted only when the following criteria were met: < 5ppm differences between measured and theoretical mass (based on chemical formula), < 30 s differences between authentic standard and analyte retention times, isotope peak abundance measurements for analytes were > 90% matched to the theoretical value generated from the chemical formula. Where measured, fragmentation patterns were matched to least the base peak and two additional peak matches in the MS/MS spectrum to within 12ppm. The top 10 data directed fragmentation method was not always able to provide fragment ions for all ions measured in the MS 1 spectrum.

Data analysis

Principal Component Analysis (PCA) was performed using Progenesis QI. Fold change, % CV and p values were generated automatically in progenesis QI and verified manually using a normalized abundance output and Excel. Heatmaps were generated manually

using the verified fold-change output. p values were generated using ANOVA (independent conditions). Statistical differences were annotated according to the scale *$p < 0.05$, **$p < 0.01$, ***$p < 0.001$.

Unsupervised clustering analysis was performed using the ClustVis web tool (https://biit.cs.ut.ee/clustvis/).

AMPK ELISA

Monocytes were differentiated in the presence of M-CSF and butyrate or left untreated for 5 days. For quantification of pAMPK phosphorylation 100,000 macrophages were plated in flat bottom plates and AMPK quantification was performed at baseline after cell lysis as per the manufactures protocol (KHO0651; Thermo scientific).

ANNEXIN V AND 7AAD STAINING

For the Annexin V and 7AAD staining, 250,000 cells were harvested after 5 days of differentiation. In the case of *Salmonella* infection, cells were infected at an MOI of 10 for 1 h prior to staining. Annexin V and 7AAD staining was performed as per the manufactures protocol.

MEASUREMENT OF REACTIVE OXYGEN SPECIES

Production of reactive oxygen species by macrophages was evaluated with the chemiluminescence probe L-012 (100 μM, Wako laboratories, Japan) in opaque white 96-well plates. Cells were activated with 100ng/mL of PMA or infected with *Salmonella* (MOI 10) for 30 min. The luminescence was recorded every 2 min for 90 min with a plate reader (FLUOstar OPTIMA, BMG labtech). For the flow cytometric dihydrorhodamine (DHR) assay, macrophages were treated with DHR (2.5 μg/mL) and stimulated with/without 100 ng/mL PMA and analyzed by flow cytometry.

CONFOCAL MICROSCOPY

Quantification of GFP positive bacteria and *Salmonella*-associated LC3 was performed by confocal microscopy. Control and butyrate treated macrophages ($1x10^5$) were seeded in 8-well chamber slides (Sarstedt) and were infected with GFP *Salmonella* Typhimurium at an MOI of 20 for 1 h. Cells were then treated for an additional 1 h with gentamicin (100μg/ml) to kill extracelluar *Salmonella*. Cells were fixed with 2% paraformaldehyde and then permeabilized with 0.1% Triton X-100 for 10 min. Fixed and permeabilized cells were stained with anti-LC3 (clone PM036, MBL) and secondary antibodies (Alexa Fluor 568 goat anti-rabbit IgG, Life technologies). Cellular and bacterial DNA was stained with DAPI. Finally, images of macrophages were acquired as z stacks of multiple sections collected at 0.5 μm intervals at 63x magnification with a Zeiss 510 or 780 inverted confocal microscope (ZEN2009 or ZEN2011 software). A minimum of 100 infected cells were evaluated and quantified with ImageJ software. For the microscopy quantification of *Salmonella* degradation, bacteria were classified into GFP^{bright} $LC3^{negative/dim}$ (i.e., early stage *Salmonella* infection with intact GFP signal) and GFPdimLC3bright (LC3 coated *Salmonella* with quenched or degraded GFP signal). In addition there are intermediate stages of $GFP^{dim}LC3^{dim}$ (Intermediate phase) and $GFP^{negative}LC3^{negative}$ *Salmonella* (end stage degradation with bacterial DNA remnant). DAPI stain was used to identify intracellular bacteria. The microscopy acquisition setting was used to identify GFP^{bright} and $LC3^{bright}$ bacteria whereas the enhanced brightness setting of each image was used to confirm the bacterial DNA content and to identify intermediate stages.

FLOW CYTOMETRY

Phenotyping and characterization of activation markers of human monocytes-derived macrophages were performed by flow cytometry. Cells were harvested, washed and counted before being incubated for 20 min at 4°C in PBS with 5% BSA containing the following surface antibodies: CD14 (clone M5E2) (Biolegend), CD11c (clone B-Ly6), HLA-DR (G46-6) (BD Biosciences) and fixable viability dye (eBioscience). Following surface staining, cells were fixed and permeabilized with the Cytofix/Cytoperm Fixation/Permeabilization Solution Kit (BD Biosciences) according to the manufacturer's instructions. Cells were intra-cellularly stained with S100A8 (clone CF-145, eBioscience), S100A9 (clone MRP1H9, Biolegend), phospho-S6 (ser235, ser236) (clone cupk43k, eBioscience), acetyl-histone H3 (lys27) (clone D5E4) (New England biolabs) and tri-methyl-histone H3 (lys27) (clone C36B11) (New England biolabs). LC3-turnover was assessed by flow cytometry with the Autophagy Detection Reagent according to manufacturer's instructions (Merck Millipore). GLUT1 receptor expression was quantified by flow cytometry (n = 4 healthy donors). 250,000 macrophages were surface stained in the flow cytometry staining buffer for 20 min on ice. Glucose uptake was quantified using 2-NBDG, a fluorescent glucose analog. Cells were incubated with 10μM 2-NBDG in RPMI + 10% FCS media at 37°C. All cells were acquired on a LSRII or a Fortessa (BD Biosciences). All analysis was performed using FlowJo software (Tree Star, Ashland, OR).

$CD45^+CD3^+CD4^+FOXP3^+$ T CELLS QUANTIFICATION

Cells were isolated as described in Methods (Isolation of mouse colonic macrophages). Staining was performed on uninfected mice or 48 h after *Salmonella* infection. Butyrate-treated mice received butyrate 5 days before and during infection in the drinking water. Lamina propria and spleen $CD45^+CD3^+CD4^+FOXP3^+$ T cells were analysed by flow cytometry.

ANALYSIS OF SINGLE-CELL TRANSCRIPTOMICS DATA

Single-cell RNA-sequencing libraries were generated using the 10x Genomics Single Cell 3′ Solution (version 2) and subjected to Illumina sequencing (HiSeq 4000). The computational workflow used to analyze the 10x Genomics data is available at https://github.com/sansomlab/tenx. Briefly, reads were aligned using 10x Genomics Cell Ranger pipeline (version 2.0.2) and human reference sequences (version 1.2.0). To circumvent known index-hopping issues with the HiSeq 4000 platform (Sinha et al., 2017), cell barcodes common to more than one sample were removed from the aggregated count matrix. UMI counts were randomly down-sampled so as to normalize the median number of per-cell counts between the samples. Data were then processed using the Seurat CRAN package (version 2.2.0). Cells with > 20% mitochondrial reads or fewer than 500 genes were excluded from the analysis. Per-cell counts were normalized, scaled and the effects of total UMI count, percentage of mitochondrial UMI count, and donor (within the control and butyrate conditions) regressed out. Cells from the two conditions were then aligned using an approach based on canonical correlation analysis (Butler & Satija, 2018). We retained the first 13 CCA components, and discarded cells for which the variance explained by CCA was < 2-fold (i.e., ratio < 0.5) of that observed with PCA. Clusters of cells were identified using the FindClusters function of the Seurat package (original Louvain algorithm, resolution = 0.3) and visualized by t-Distributed Stochastic Neighbor Embedding (t-SNE) projection (perplexity = 20, Figure 3A) (Van der Maaten and Hinton, 2008). For each cluster of cells, markers were identified using the MAST test (Finak et al., 2015) as implemented in the Seurat package (default parameters; Table S1). Conserved cluster markers (Figure 3C) were identified as those achieving a maximum BH adjusted P value of < 0.05 when tested within each of the samples separately. Gene set enrichment analysis (Figure 3E) was performed using Fisher's Exact Test (FET), Biological Process gene sets obtained from Gene Ontology (GO) databases (Ashburner et al., 2000; Kanehisa and Goto, 2000; Liberzon et al., 2011; Subramanian et al., 2005), and a gene universe that comprised of genes expressed in the differentiated Macrophages (n = 11,218). To identify genes differentially expressed between control and butyrate macrophages, we first summed the counts for each samples' cells within each of the clusters. The DESeq2 package (Love et al., 2014) was then used to model the replicated, paired design and to perform a test for differential gene expression between control and butyrate macrophages within each of the clusters. The accession number for the single cell sequencing reported in this paper is Gene Expression Omnibus: GSE111049.

QUANTITATIVE PCR

RNA was isolated from macrophages, or from flow cytometry-sorted cells using the RNeasy Mini kit (QIAGEN, Manchester, UK) according to the manufacturer's instructions. cDNA synthesis was performed using High-Capacity cDNA Reverse Transcriptase (Applied Biosystems, Life Technologies, Paisley, UK). qPCR were performed using TaqMan gene expression assays (Life Technologies) and TaqMan Universal PCR master mix. qPCR were run on the CFX96 detection system (Bio-Rad Laboratories, Hemel Hempstead, UK) and gene expression for each sample were normalized to RPLPO for human reference gene or HPRT for mouse reference gene and the differences were determined using the $2\Delta C(t)$ calculation.

QUANTITATION OF CYTOKINE SECRETION

Calprotectin was measured by ELISA (Biolegend) in the culture supernatants of control and butyrate macrophages after 5 days of differentiation by ELISA accordingly to the manufacturer's instruction and normalized to the cell count.

TRANSFECTION OF PRIMARY HUMAN MACROPHAGES

The Accell SMARTpool siRNA were used to target human *S100A8*, *S100A9* and *HDAC3* for siRNA-mediated gene silencing in macrophages according to the manufacturer's instructions (Dharmacon).

IMMUNOBLOT

Cells were lysed and the protein extract was quantified by BCA (Thermo Scientific). Equal amounts of total cell lysates (20 μg) was run on SDS-PAGE gels (4%–20%) (Bio-Rad), followed by transfer to polyvinylidene difluoride membranes. Proteins were detected using primary antibodies against LC3B (D11, Cell signaling), β-actin (13E5, Cell signaling), phospho-S6 ribosomal protein (Ser235 and/or 236) (D57.2.2E, Cell signaling), S100A8 (EPR3554, Abcam), acetylated-H3 (ab47915, Abcam) and acetylated-H4 (EPR16606, Abcam), P62 lck (clone 3/P62, BD bioscience) and detected using HRP-conjugated secondary antibodies.

ISOLATION OF LYMPHOID AND MYELOID POPULATIONS FROM HUMAN BLOOD

PBMC from healthy donors were isolated as detailed above and stained for surface markers in PBS with 0.1% bovine serum albumin buffer allowing MACS enrichment and FACS sorting. $CD4^+$ T cells were enriched by depleting $CD8^+$, $CD19^+$, $CD56^+$ and $CD14^+$ cells by MACS and then stained using anti-CD4, anti-CD45RA and anti-CD45RO to sort naive $CD4^+CD45RA^+$ and memory $CD4^+CD45RO^+$ T cells by FACS. B cells, $CD8^+$ T cells and NK cells were sortedby FACS using anti-$CD19^+$ for B cells, anti-CD8 for $CD8^+$ T cells or anti-$CD56^+$ for NK cells. $CD141^{high}$ and $CD1c^+$ DC populations were enriched by depleting $CD3^+$, $CD19^+$, $CD56^+$ and $CD14^+$ cells by MACS and FACS sorted to isolate HLA-DR^{high} $CD11c^{low}$ $CD141^{high}$ for $CD141^+$ DC or HLA-DR^{high} $CD11c^{high}$ $CD141^-$ $CD1c^+$ for $CD1c^+$DC. All cells were sorted using an AriaIII flow cytometer (BD Biosciences) to a purity of > 95%. $CD14^+$ monocytes were obtained by anti-CD14 bead enrichment using MACS sort.

EXPERIMENTAL MODEL AND SUBJECT DETAILS

Animals were bred and maintained under specific pathogen-free conditions. Animal experiments were performed in accordance with the UK Scientific Procedures Act (1986) under a Project License (PPL) authorised by the UK Home Office. 8- to 12-week-old inbred female C57BL/6J were used for experiments and were bred and maintained at the University of Oxford. Mice were routinely screened for the absence of pathogens, and were kept in individually ventilated cages with environmental enrichment. For experiments involving infection and/or butyrate administration, all mice in individual cages received the same treatment. To reduce cage effects each treatment was given to more than one cage in each experiment.

SODIUM BUTYRATE TREATMENT AND INFECTION

Mice received either saline, as a control, or sodium butyrate (150mM final concentration) (Sigma-Aldrich, St. Louis, MO) in their drinking water for 7 days before the sort of colonic macrophages at steady state. In case of *Salmonella Typhimurium 1344 def AroA* and *Citrobacter rodentium* infection, mice received saline as a control or sodium butyrate in drinking water for 5 days and 3 days respectively before infection. For the infection, mice were intragastrically gavaged with nalidixic acid resistant Citrobacter rodentium (ICC169) ($1x10^9$) or Salmonella Typhimurium ($1x10^9$). Mice weight was monitored throughout the duration of experiment (7 days for *Citrobacter rodentium* and 2 days for *Salmonella Typhimurium*).

HISTOLOGY SCORE

Samples of the proximal colon were collected and fixed in buffered 10% of 36% formalin solution to assess the severity of colitis. Haematoxylin and eosin (H&E) staining was performed on 4–5 μm paraffin-embedded sections and inflammation was assessed using previously published criteria (Izcue et al., 2008). Each sample was graded semiquantitatively based on the following features: cellular infiltration of the lamina propria, epithelial hyperplasia and goblet cell depletion, percentage of the section affected, and markers of severe inflammation (submucosal inflammation, crypt abscesses) from 0 to 4 in each of the 4 parameters. Samples were scored by two individuals blinded and scores for each criterion were added to give an overall score for each sample of 0–12.

ISOLATION OF MOUSE COLONIC MACROPHAGES

Colons from 10 mice gavaged with PBS and with sodium butyrate were prepared as described (Arnold et al., 2016). Briefly colons were opened, lumenal content removed and washed, and tissue cut into pieces. Pieces were washed twice in RPMI-1640 medium supplemented with 5% fetal bovine serum and 5mM EDTA at 37°C with shaking to remove epithelial cells. Tissue was then digested in RPMI medium containing 1mg/mL type VIII collagenase (Sigma-Aldrich, Gillingham, UK), 0.5mg/mL DNase I and 5% fetal bovine serum. Cells were then layered on a 40/80% Percoll gradient, centrifuged, and the colonic leukocytes at the gradient interface were recovered and were stained in phosphate-buffered saline with 0.1% bovine serum albumin buffer with a combination of the following antibodies: CD45 (30-F11), CD11c (N418), MHCII (M5/114.15.2), F4/80 (BM8); CD103 (M290), CD11b (M1/70), CD64 (X54-5/7.1). Before the sort, DAPI was added to differentiate viable from dead cells. Macrophages were defined as ($CD11b^+$ $CD64^+$ $F4/80^+$ $MHCII^+$ $CD103^-$) positive. All cells were sorted on a AriaIII (BD Biosciences) flow cytometer to a purity of > 95%.

BONE MARROW DERIVED MACROPHAGES

In brief, bone marrow was extracted from the femur of mice, washed, cells were counted and seeded at the concentration of $7x10^6$ per 10cm dish in 10 mL of RPMI supplemented with 50 ng/mL murine M-CSF with 10% FCS (Sigma-Aldrich), 100 U/mL penicillin, 100 μg/mL streptomycin, 30 mM HEPES for 7 days with or without butyrate (1mM).

PHAGOCYTOSIS ASSAY

Macrophage phagocytic activity was measured by two different methods: Firstly by using *Escherichia coli* (K-12 strain) BioParticles™, Alexa Fluor™ 488 conjugate as per manufracture's instruction; Secondly, macrophages were infected with GFP-*Salmonella* and incubated for indicated time (15 min, 30 min, 45 min, 60 min). Cells were washed and the fluorescence due to *Salmonella* attached to the outside of the cells was quenched with trypan blue solution (4%). The fluorescence was evaluated by flow cytometry.

STATISTICS

Depending on the dataset, a Mann–Whitney nonparametric-test or Kruskal Wallis test (one-way Anova analysis) was used to calculate significance between groups. Graph generation and statistical analyses were performed using Prism version 5.0d software (GraphPad, La Jolla, CA). Differences were considered statistically significant when $p < 0.05$.

DATA AND SOFTWARE AVAILABILITY

The accession number for the single cell sequencing reported in this paper is Gene Expression Omnibus: GSE111049. This accession number will be accessible to readers upon publication of the manuscript.

Immunity, Volume *50*

Supplemental Information

The Short Chain Fatty Acid Butyrate Imprints an Antimicrobial Program in Macrophages

Julie Schulthess, Sumeet Pandey, Melania Capitani, Kevin C. Rue-Albrecht, Isabelle Arnold, Fanny Franchini, Agnieszka Chomka, Nicholas E. Ilott, Daniel G.W. Johnston, Elisabete Pires, James McCullagh, Stephen N. Sansom, Carolina V. Arancibia-Cárcamo, Holm H. Uhlig, and Fiona Powrie

Supplemental Figures

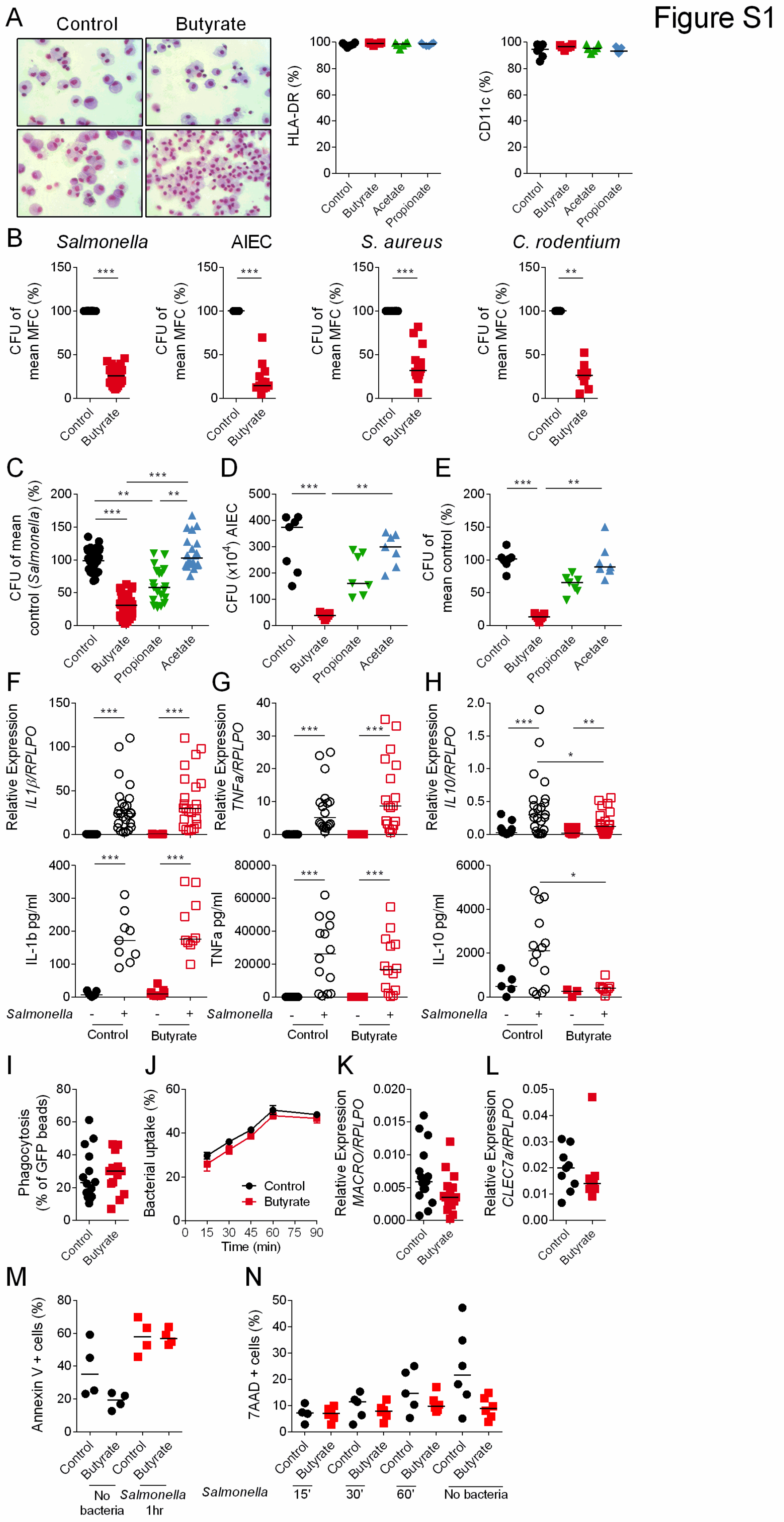

Figure S1: Increased antimicrobial activity by Mϕ differentiated in the presence of SCFA (Figure 1)

(A) Morphology and quantification of CD11c and HLA-DR expression on Control (left) and Butyrate (right) macrophages visualised by GIEMSA staining of cytospin preparations (X100) and FACS. Each dot represents one independent donor.

(B) Control and butyrate differentiated macrophages were infected for 1 hour with *Salmonella*, *AIEC*, *S. aureus* and *C. rodentium* followed by gentamicin treatment for 2 hours before cell lysis. Values are represented as % CFU of mean control macrophages.

(C-E) Gentamicin protection assay on Control, Butyrate, Acetate and Propionate differentiate macrophages infected with Salmonella (C) and (D) AIEC. Values are represented as % CFU of mean control macrophages or as absolute CFU count (E).

(F-H) Relative mRNA and protein expression of *IL1b* (F), *TNFa* (G) and *IL10* (H) by Control and Butyrate macrophages, at steady state and after 3 hours of *Salmonella* infection.

(I, J) Mϕ phagocytosis rates were assessed using FITC beads coated with human IgG on a flow cytometry-based assay and (J) bacterial uptake was assessed using non-opsonised GFP-*Salmonella* over 90 min.

(K-L) Relative mRNA expression of *MARCO* (K) and *CLEC7A* (L) in Control and Butyrate macrophages.

(M-N) Annexin V staining (n=4) (M) and 7AAD staining (N) in Control and Butyrate macrophages (n=4-6).

Each dot represents a healthy donor. Mann-Whitney U test was performed to investigate significant differences. Each dot represents one independent donor. Statistical significance was determined using Mann-Whitney U test *p<0.05, **p<0.01 and ***p<0.001.

Figure S2

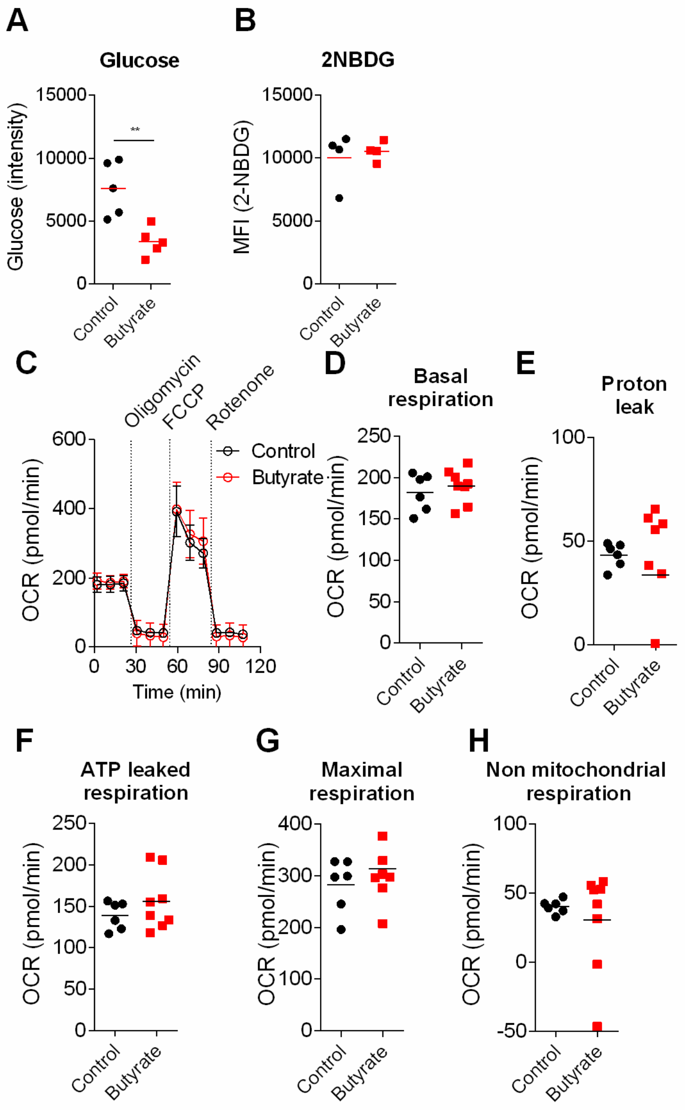

Figure S2: Oxygen consumption rate is similar in control and butyrate-treated macrophages (Figure 2)

(A) Intracellular glucose amounts were determined by Liquid chromatography /Mass Spectrometry (n=5).

(B) Uptake of 2-NBDG , a fluorescent glucose analog measured by FACS (n=4).

(C-H) Oxygen consumption rate of control and butyrate-treated macrophages after oligomycin, Carbonyl cyanide-*4*-(trifluoromethoxy) phenylhydrazone (FCCP) and rotenone treatment over time as determined by seahorse mito-stress test (C). Individual components of this assay including: Basal respiration (D), proton leak (E), adenosine tri-phosphate (ATP) leaked respiration (F), maximal respiration (G), Non mitochondrial respiration (H) is presented. Experiments were performed on each

donor with 8 technical replicates. Experiments with 3 different donors yielded similar results. Statistical significance was determined using **$p<0.01$; Mann-Whitney U test.

Figure S3

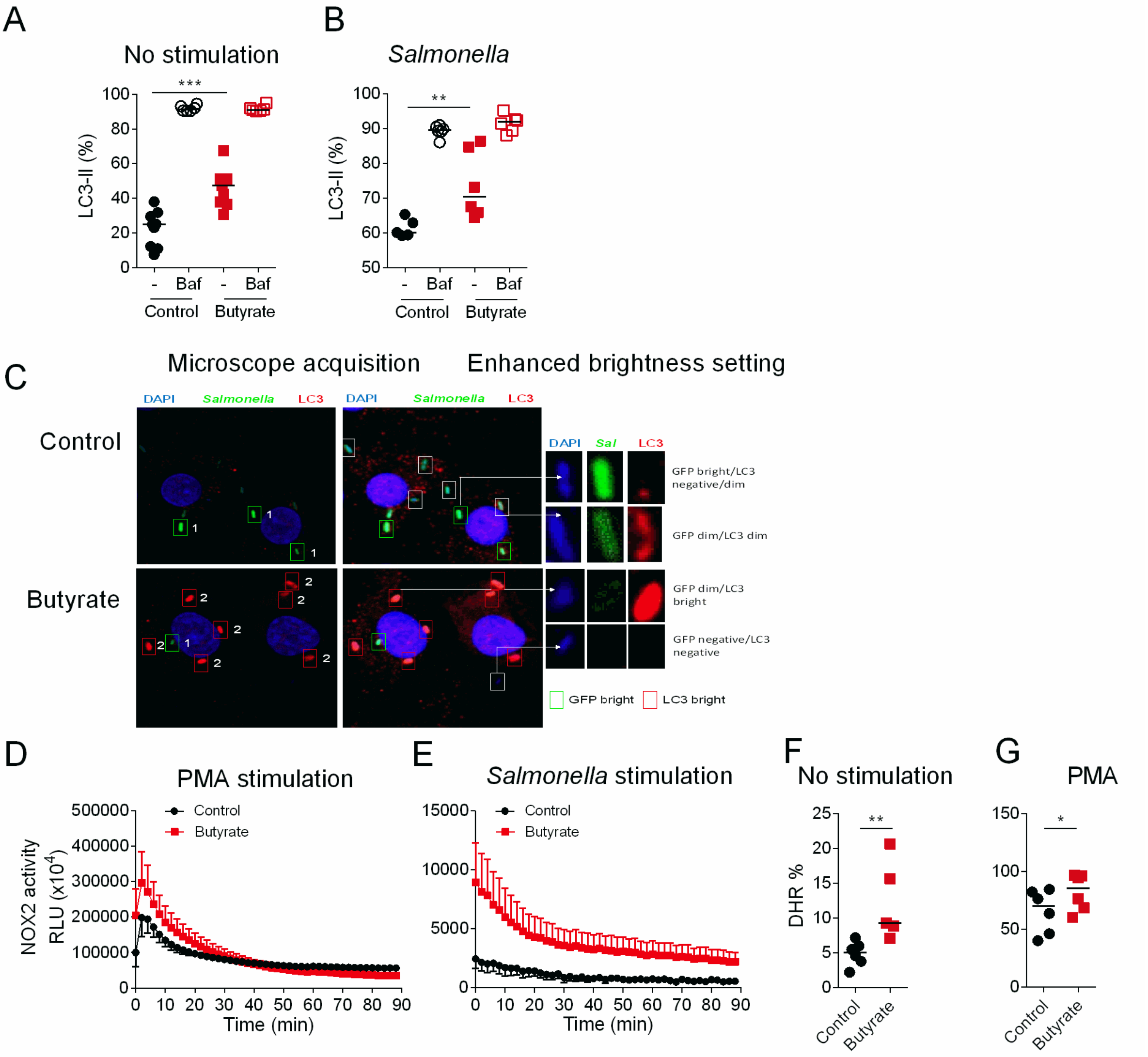

Figure S3 Increase of LC3 coated bacteria and ROS production in butyrate macrophages (Figure 2)

(A, B) Percentage LC3-II expression by flow cytometry at steady state (A) or after 1 hour infection with *Salmonella* (B) in Control and Butyrate macrophages. In the control conditions, cells are treated with 75nM of Bafilomycin A1 (baf) (positive control) for 2 hour before the LC3 staining.

(C) Representative confocal microscopy image of Control and Butyrate macrophages showing different stages of *Salmonella* degradation with increased LC3 coating in Butyrate macrophages (example quantification corresponding to the images provided in Fig. 2L). Control and Butyrate macrophages were infected with GFP-*Salmonella* and stained with LC3 and DAPI. For the microscopy quantification of *Salmonella* degradation, bacteria were classified into GFP^{bright} LC3$^{negative/dim}$ (i.e. early stage *Salmonella infection with intact gfp* signal) and GFP^{dim}LC3^{bright} (LC3 coated *Salmonella* with quenched or degraded gfp signal). In addition there are intermediate stages of GFP^{dim}LC3^{dim} (Intermediate phase)

and GFP^{negative}LC3 ^{negative} *Salmonella* (end stage degradation with bacterial DNA remnant). DAPI stain was used to identify intracellular bacteria. The microscopy acquisition setting was used to identify GFP^{bright} and LC3^{bright} bacteria whereas the enhanced brightness setting of each image was used to confirm the bacterial DNA content and to identify intermediate stages.

(D, E) ROS produced by NOX2 was quantified by a chemi-luminescence based L0-12 assay in Control and Butyrate macrophages stimulated with or without PMA (100ng/ml) (D) or infected by *Salmonella* at MOI 10 for 30 min (E) before evaluating NOX2 activity. Data represent the mean of 4 independent experiments from 3 donors.

(F, G) Intracellular reactive oxygen species evaluated with dihydrorhodamine (DHR) FACS in Control and Butyrate macrophages at steady state (F) or after PMA stimulation for 15 min (G). Each dot represents one independent donor.

Statistical significance was determined using Mann-Whitney U test *$p<0.05$, **$p<0.01$ and ***$p<0.001$.

Figure S4

A

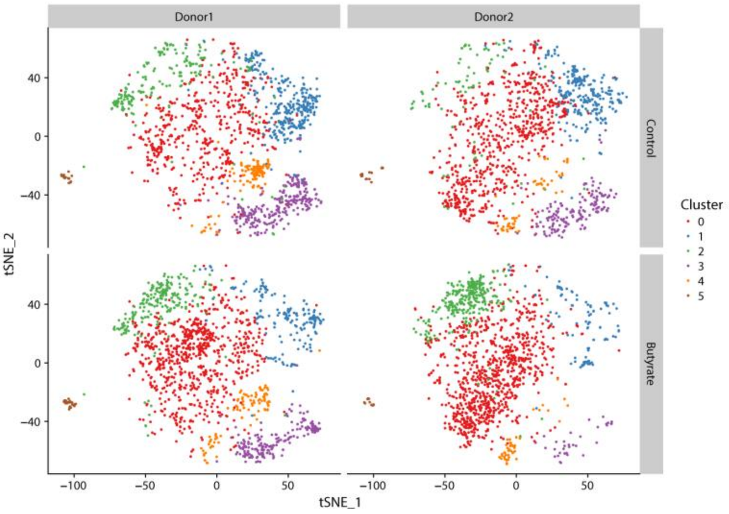

B

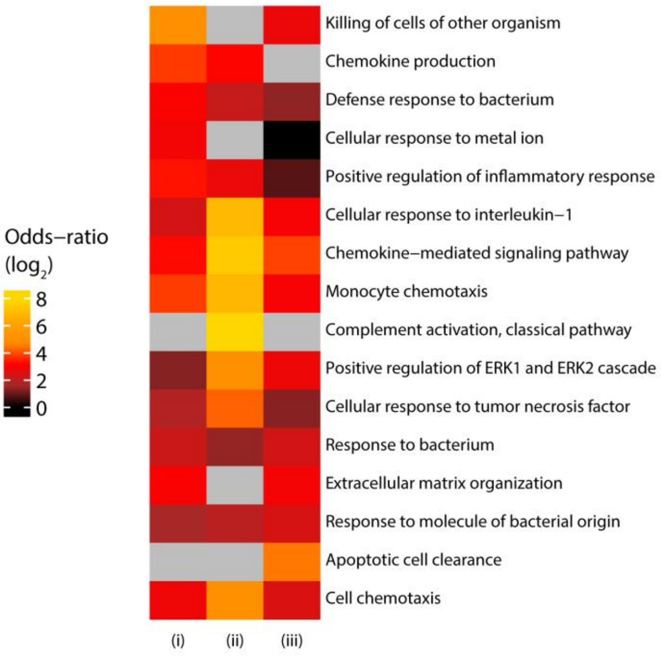

Figure S4: Impact of butyrate across subpopulations of differentiated macrophages (Figure 3)

(A) The t-SNE projection shown in Fig 4, deconvoluted to reveal the distribution of cells from the four

individual samples. A noticeable increase of cells in the lysosomal (cluster 0) and antimicrobial (cluster 2) compartments can be observed.

(B) Comparative heat map of log_2 odds-ratio for over-representation of biological pathways (GO) in genes (i) commonly up-regulated by butyrate across the entire population of differentiated macrophages, (ii) specifically down-regulated in cluster 2 (antimicrobial Macrophages), and (iii) globally down-regulated by butyrate across the entire population of differentiated Macrophages.

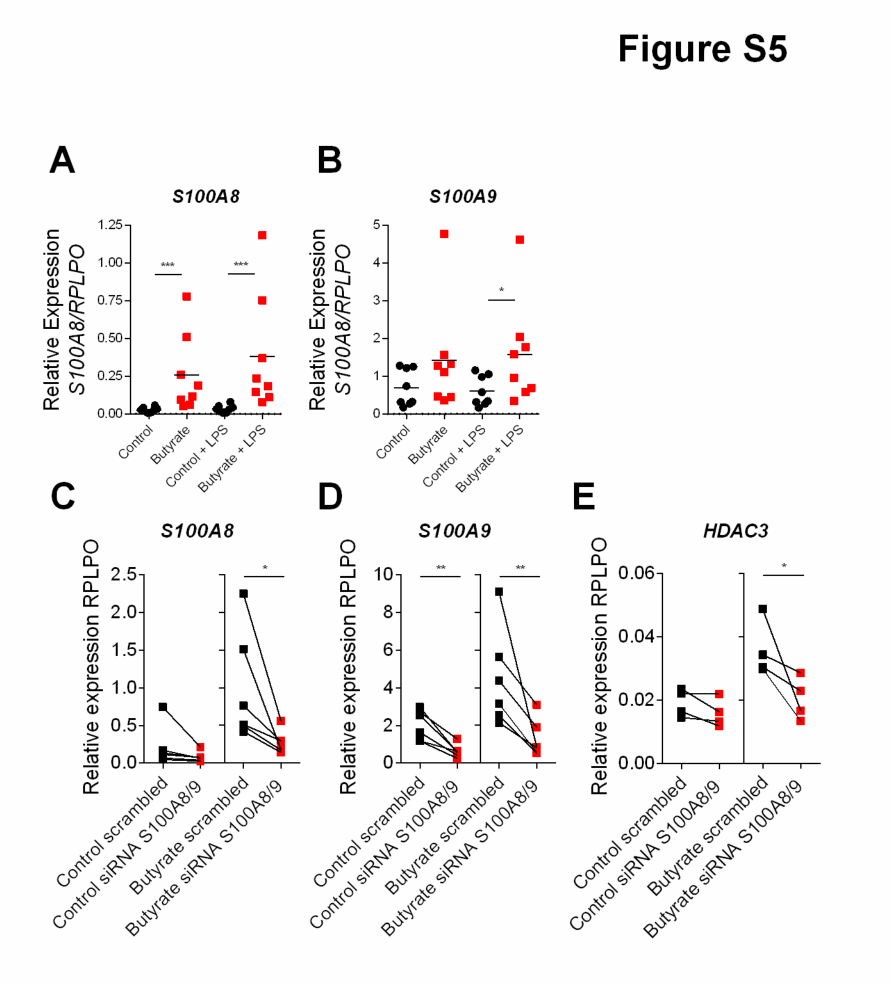

Figure S5: *S100A8*, *S100A9* and *HDAC3* mRNA expression in control and butyrate-treated macrophages. (Figure 4 and 5)

(A-B) *S100A8* (A) and *S100A9* (B) mRNA expression was measured by qPCR in control and butyrate-treated macrophages in the presence or absence of LPS. Each dot represents one donor (n=8).

(C-E) *S100A8* (C) *S100A9* (D) and HDAC3 (E) mRNA expression was measured by qPCR. Each dot represents one donors (n=6).

Statistical significance was determined using Mann-Whitney U test *p<0.05, **p< 0.01, ***p<0.001.

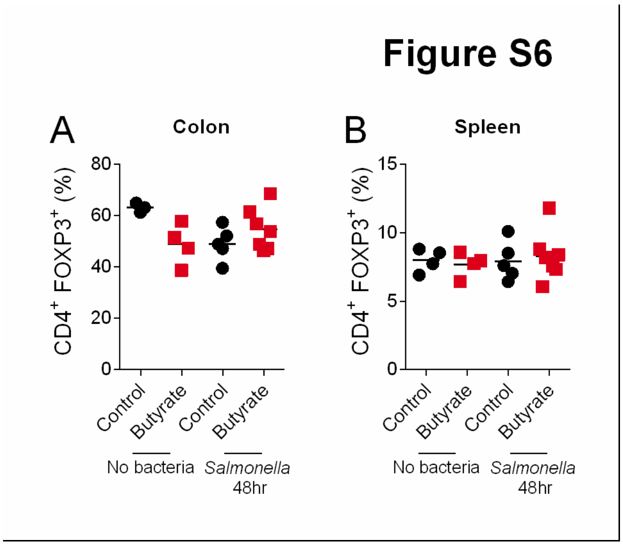

Figure S6: $CD4^{+}FOXP3^{+}$ cells do not show any change in Control and butyrate treated mice. (Figure 6)

(A-B) Percent of $CD4^{+}FOXP3^{+}$ cells in the colon (A) and (B) spleen. Colonic lamina propria and splenic $CD45^{+}CD3^{+}CD4^{+}FOXP3^{+}$ T cells analysed by flow cytometry in uninfected mice or 48hours after *Salmonella* infection. Butyrate-treated mice received butyrate 5 days before and during infection in the drinking water.

Supplementary Tables

Table S1: Cluster marker genes common to both control and butyrate macrophages

The table contains the positive and negative marker genes identified for each of the clusters of macrophages discovered by the single-cell RNA-sequencing analysis (Fig 3A). Only marker genes that achieved significance in all separate, per-sample tests (BH adjusted p-value < 0.05) are listed.

Table S2: Genes differentially expressed between between control and butyrate macrophages within each cluster of differentiated macrophages.

This table contains the lists of genes differentially expressed by between control and butyrate macrophages within each of the clusters of differentiated (non-cell cycle associated) macrophages (see Methods).

Table S3: Gene Ontology categories enriched in genes differentially expressed between control and butyrate macrophages

The table contains the details of the Gene Ontology (Biological Process) categories found to be signficantly enriched in each of the three groups of genes differentially expressed between control and butyrate macrophages (Fig 3E).
